# Straightforward Creation of Possibly Prebiotic Complex Mixtures of Thiol-Rich Peptides

**DOI:** 10.3390/life13040983

**Published:** 2023-04-10

**Authors:** Ibrahim Shalayel, Naoual Leqraa, Véronique Blandin, Yannick Vallée

**Affiliations:** 1 Université Grenoble Alpes, TIMC-IMAG, CNRS, F-38000 Grenoble, France; ibrahim.shalayel@univ-grenoble-alpes.fr; 2 Université Grenoble Alpes, DCM, CNRS, F-38000 Grenoble, France; naoualleqraa@gmail.com (N.L.); veronique.blandin@univ-grenoble-alpes.fr (V.B.)

**Keywords:** prebiotic chemistry, abiogenesis, cysteine, nitriles, peptides, macrocycles

## Abstract

At the origin of life, extremely diverse mixtures of oligomers and polymers could be obtained from relatively simple molecular bricks. Here, we present an example of the polymerization of two amidonitriles derived from cysteine, Cys-Ala-CN and Cys-Met-CN. The thiol function in a molecule adds onto the nitrile group of another one, allowing efficient condensation reactions and making available an extensive range of polymers containing amide bonds and/or five-membered heterocycles, namely thiazolines. Macrocycles were also identified, the biggest one containing sixteen residues (cyclo(Cys-Met)_8_). MALDI-TOF mass spectrometry was used to identify all the present species. What these examples show is that complex mixtures are likely to have formed on the primitive Earth and that, ultimately, the selection that must have followed may have been an even more crucial step towards life than the synthesis of the pre-biological species themselves.

## 1. Introduction

The concept of life’s origin is related to the chemistry of primitive molecules and minerals [1,2,3,4,5]. The early Earth [6] was a complex reactor in which a continuous flow of numerous chemical reactions occurred. The variety of possible reagents and small organic molecules was huge [7]. Their reactions produced masses of larger molecules, oligomers, and polymers, which could lead to a crowded situation [8]. In such a messy environment, the way to life was not a straight road but an entanglement of uncertain paths, dead ends, backtracking, narrow passes, and roundabouts.

Even a very simple starting material, such as formaldehyde [9], may lead to an entire library of derivatives, including hydroxy acetic acid, glycine, *N*-substituted glycines, and hydroxyacetaldehyde, hence providing lots of sugars [10] and various nitrogen heterocycles. The reaction of any given aldehyde with ammonia and cyanide anion would give an aminonitrile, which would be hydrolysed to an aminoamide and then to an amino acid [11,12]. Amino acids would condense, delivering messy mixtures of peptides, including not only linear ones but also branched peptides thanks to diamino acids (e.g., lysine). However, such polymerization would be counterbalanced by amide bond hydrolysis, so there would always be monomers and short chains, and the most stable sequences would be favored in long chains. Esters would be formed from serine residues, adding ester functions to the polyamide main chains, and thioesters may also be formed from cysteine residues (compared to non-ribosomal aminoacyl transferases nowadays) [13]. In addition, nothing precludes monomeric amino acids from reacting with other types of monomers. They may react with sugars at the anomeric position or at other positions (as they do currently at the 3′ and 2′ positions of the terminal adenosine of t-RNAs).They may also react with many other acids to give diverse amides (different from peptides), just as aspartic acid takes part in the de novo synthesis of inosine, for instance [14]. Many heterocycles would also be present [15,16]. Thus, starting from a great multiplicity of components, an incredibly messy mixture of molecules would have formed—most of which were not relevant at all to the development of life. 

**Figure 1:**
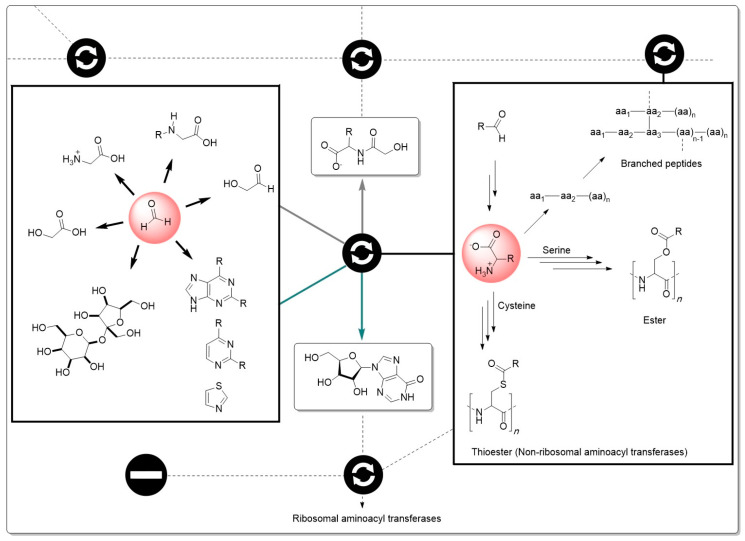
Part of the “prebiotic mess” from which life originated. Traffic circle sign: chemical link between groups of reactions; no entry sign: non-efficient processes. Some non-represented reagents are necessary, such as HCN and ammonia.

Even if today’s cellular metabolism may seem (and in fact is) incredibly complex and diverse—with millions of elementary reactions taking place every second in the cells that constitute, for example, a human body—this is in fact only a small sample of what chemistry (both organic and inorganic) would make possible. What from afar may look like an almost infinite sum of happy accidents is in fact meticulously governed and follows invariable rules. Chemical selection gradually transformed the “prebiotic mess” into biology (Figure 1). As one selection factor, certain types of molecules may have been more prone than others to transforming into more complex structures while carrying along enough chemical reactivity features to be able to evolve again and again, until the structure would gain a function in its environment. Remnants of these molecules’ characteristics would likely be seen in today’s biological machinery.

**Scheme 1:**
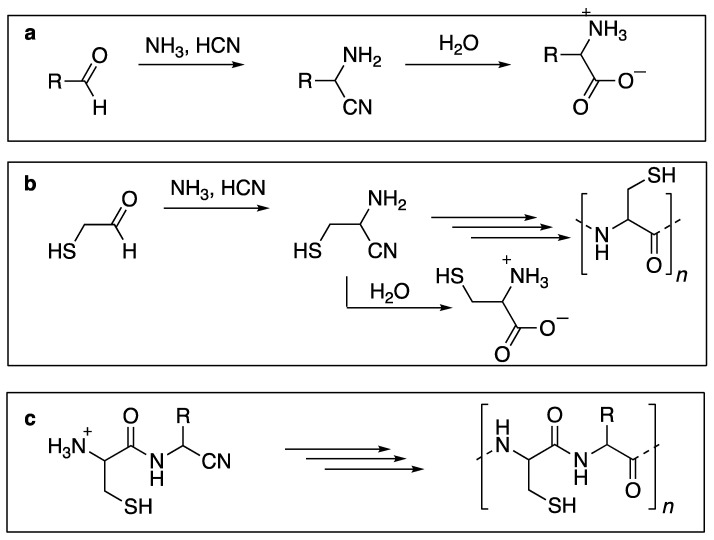
(**a**) Strecker reaction, (**b**) Strecker reaction applied to the synthesis of cysteine and polycysteine peptides, (**c**) this work (R = Me, CH_2_CH_2_SMe).

Looking into proteins, cysteine residues with their thiol functional group shows a remarkable tendency for their presence in catalytic sites [17]. The presence of thiol groups allows protein folding and redox reactions via the formation of disulfide bonds. As they are part of iron–sulfur clusters, they are key players in the formation of metalloproteins, such as ferredoxins (four cysteine residues involved), and in zinc fingers (in which up to six cysteine units are involved), both of which are important in protein–protein and DNA (or RNA)–protein interactions. In view of the importance of thiols, the hypothesis that cysteine has been integrated into the primitive peptide chains is persuasive [18]. Plausible prebiotic syntheses of cysteine have been described [19,20]. The next question is: how were cysteine residues able to assemble with the other amino acids to form thiol-containing peptides that grow and finally gain a function? It is possible that both the formation and polymerization of amino acid units were related and synchronized while in harmony with the geochemical environment. Hydrogen cyanide may have played a crucial role in such chemistry. HCN is widespread in the cosmos and seen in different areas, such as near carbon stars, proto-planetary nebulae, and comets [21,22]. Therefore, it is likely that HCN existed on the early Earth. This would have given a central role to the Strecker reaction (Scheme 1a) in the synthesis of amino acids, including cysteine (Scheme 1b). From the aminonitrile intermediate of the Strecker reaction, dipeptides containing terminal nitrile function may have been formed, including Cys-AA-CN (where AA stands for any amino acid) [23]. In this paper, we describe our results from the condensation of two representative examples of these aminothiol-containing amidonitriles and demonstrate that they readily polymerize, thereby giving highly complex mixtures of thiol-rich polypeptides (Scheme 1c).

## 2. Materials and Methods

Products were observed in reaction mixtures by NMR spectroscopy (^1^H and ^13^C) and mass spectrometry. NMR-monitored reactions were run in D_2_O solutions in NMR tubes. The NMR apparatus was Bruker Avance III 400 or 500. For the mass experiments, H_2_O was used as the solvent. High-resolution mass spectra were recorded on a Waters G2-S Q-TOF mass spectrometer or on a LTQ Orbitrap XL (Thermo Scientific, Waltham, MA, USA) spectrometer. Low-resolution ESI analysis was performed on an Amazon speed (Brucker Daltonics, Billerica, MA, USA) IonTrap spectrometer. For MALDI analysis, DMSO was used as the solvent to dissolve the obtained polymers, and DHB (2,5-Dihydroxybenzoic acid) was used as the matrix.

**Scheme 2:**
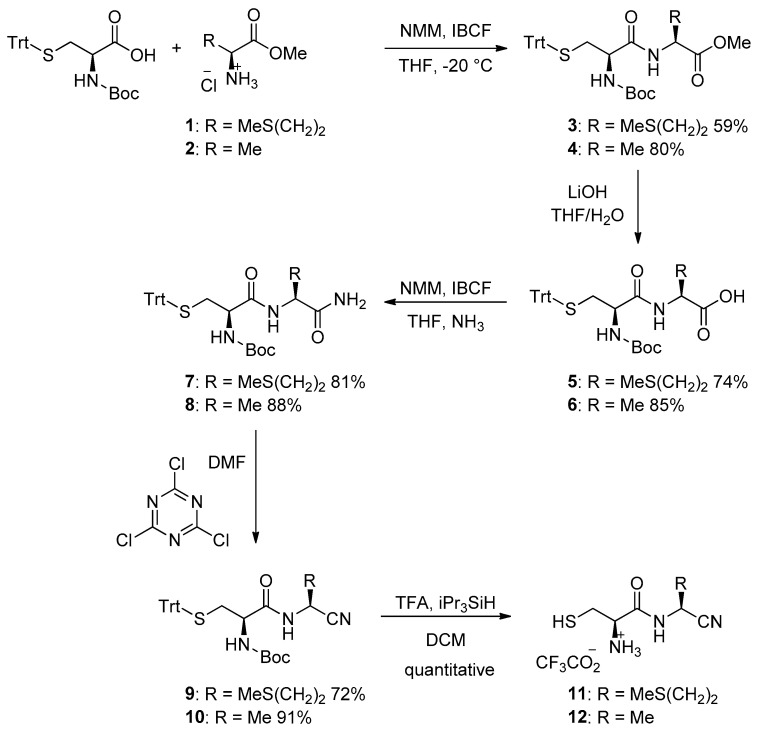
Synthetic pathway towards Cys-Met-CN **11** and Cys-Ala-CN **12**.

General synthetic pathway was used for the preparation of amidonitriles Cys-Met-CN and Cys-Ala-CN (Scheme 2).

Boc-Cys(Trt)-OH (N-(*tert*-butoxycarbonyl)-S-trityl-l-cysteine) was activated with IBCF (*iso*butyl chloroformate) in the presence of NMM (N-methyl morpholine). Then, methyl esters of, respectively, methionine **1** and alanine **2** were added to give the corresponding esters: respectively, **3** and **4**. The esters were then transformed into the corresponding acids **5** and **6**. These acids were activated with IBCF and NMM, and then gaseous ammonia was added to give the amides **7** and **8**. The obtained amides were then transformed to nitriles **9** and **10** using cyanuric chloride in DMF. The last step of the synthesis was the cleavage of Boc and Trt groups to obtain the nitriles **11** and **12**. The final salts were recovered in D_2_O for NMR studies or in H_2_O for mass analysis.

Characterization data for intermediates **7** and **8**

*tert*-butyl((*R*)-1-(((*S*)-1-amino-4-(methylthio)-1-oxobutan-2-yl)amino)-1-oxo-3-(tritylthio)propan-2-yl)carbamate (**7**)

m.p. 85.3–86.9 °C; [α]^20^_D_ –10,88 (*c* 1.00, CHCl_3_); ^1^H-NMR (400 MHz, CDCl_3_) (δ, ppm): 7.41 (d, *J* = 7.5 Hz, 6H), 7.31 (t, *J* = 7.5 Hz, 6H), 7.24 (t, *J* =7.2 Hz, 3H), 6.92 (s, 1H), 6.66 (s, 1H), 5.27 (s, 1H), 4.72 (s, 1H), 4.55–4.61 (m, 1H), 3.77 (dd, *J* = 11.3, 5.2 Hz, 1H), 2.78 (dd, *J* = 12.9, 6.7 Hz, 1H), 2.58 (dd, *J* =12.9, 4.9 Hz, 1H), 2.11–2.23 (m, 1H), 2.04 (s, 1H), 1.95–2.10 (m, 1H), 1.42 (s, 9H); ^13^C-NMR (100 MHz, CDCl_3_) (δ, ppm): 173.23, 170.52, 155.84, 144.20, 129.48, 128.18, 127.06, 80.85, 67.40, 60.41, 54.13, 52.24, 33.27, 28.27, 15.19; HRMS (ESI): calcd for C_32_H_39_N_3_O_4_S_2_Na [M + Na] ^+^: 616.2280, found 616.2277.

*tert*-butyl((*R*)-1-(((*S*)-1-amino-1-oxopropan-2-yl)amino)-1-oxo-3-(tritylthio)propan-2-yl)carbamate (**8**)

m.p. 97.3–101.2 °C; [α]^20^_D_ –8.0 (*c* 0.65, CHCl_3_); ^1^H-NMR (400 MHz, CDCl_3_) (δ, ppm): 7.42 (d, *J* = 7.6 Hz, 6H), 7.30 (t, *J* = 7.5 Hz, 6H), 7.24 (t, *J* = 7.2 Hz, 3H), 6.47 (s, 1H), 6.31 (d, *J* = 7.7 Hz, 1H), 5.21 (s, 1H), 4.74 (d, *J* = 3.7 Hz, 1H), 4.43 (quin, *J* = 7.3 Hz, 1H), 3.75 (d, *J* = 4.9 Hz, 1H), 2.71 (dd, *J* = 12.8, 7.4 Hz, 1H), 2.61 (dd, *J* = 12.8, 5.3 Hz, 1H), 1.42 (s, 9H) 1.36 (d, *J* = 7.2 Hz, 3H); ^13^C-NMR (100 MHz, CDCl_3_) (δ, ppm): 174.06, 170.29, 155.89, 144.19, 129.46, 128.19, 127.07, 80.91, 67.47, 53.98, 48.57, 33.28, 28.34, 28.23, 17.55.

Preparation of nitriles **9** and **10**

General procedure: amide **7** or **8** (1 equiv.) was dissolved in DMF (~6 mL). Cyanuric chloride (1 equiv.) was added. The mixture was stirred for 1 h at rt and then quenched with water and extracted three times with EA. The organic phase was washed five times with water, dried, and evaporated under vacuum. Flash chromatography afforded the pure nitriles **9** and **10**.

*tert*-butyl((*R*)-1-(((*S*)-1-cyano-3-(methylthio)propyl)amino)-1-oxo-3-(tritylthio)propan-2-yl)carbamate (**9**)


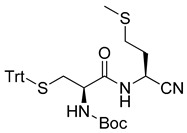


Following the general procedure, amide 7 (880 mg, 1.4 mmol) and cyanuric chloride (263 mg, 1.4 mmol) were used. The crude product was submitted to silica gel column chromatography (90–70% pentane/EA). The solvent was collected and concentrated under vacuum to give 9 as a white solid (72%).

m.p. 126.6–128.2 °C; ^1^H-NMR (400 MHz, CDCl_3_) (δ, ppm): 7.42 (d, *J* = 7.7 Hz, 6H), 7.31 (t, *J* = 7.6 Hz, 6H), 7.23 (d, *J* = 7.2 Hz, 3H), 6.73 (d, *J* = 7.5 Hz, 1H), 4.97 (d, *J* = 7.4 Hz, 1H), 4.65 (s, 1H), 3.70 (d, *J* = 5.0 Hz, 1H), 2.59 (t, *J* = 7.1 Hz, 1H), 2.53 (dd, *J* = 13.3, 5.1 Hz, 1H), 2.07 (s, 9H), 2.13–1.97 (m, 2H), 1.42 (s, 9H); ^13^C-NMR (100 MHz, CDCl_3_) (δ, ppm): 170.25, 144.23, 129.53, 128.18, 127.03, 117.67, 80.92, 67.50, 53.50, 39.48, 32.76, 32.10, 29.50, 28.23, 15.41; HRMS (ESI): calcd for C_32_H_37_N_3_O_3_S_2_Na [M + Na] ^+^: 598.2174, found 598.2180.

*tert*-butyl((*R*)-1-(((*S*)-1-cyanoethyl)amino)-1-oxo-3-(tritylthio)propan-2-yl)carbamate (**10**)


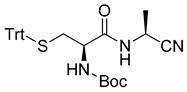


Following the general procedure, amide **8** (440 mg, 0.8 mmol) and cyanuric chloride (152 mg, 0.8 mmol) were used. The crude product was submitted to silica gel column chromatography (90–50% pentane/EA). The solvent was collected and concentrated under vacuum to give **10** as a white solid (91%).

m.p. 202.4–203.4 °C; [α]^20^_D_ –8.5 (*c* 1.00, CHCl_3_); ^1^H-NMR (400 MHz, CDCl_3_) (δ, ppm): 7.36 (d, *J* = 7.7 Hz, 6H), 7.23 (t, *J* = 7.5 Hz, 6H), 7.16 (t, *J* = 7.2 Hz, 3H), 6.48 (s, 1H), 4.72 (quin, *J* = 7.4 Hz, 1H), 4.61 (d, *J* = 5.4 Hz, 1H), 3.60–3.65 (m, 1H), 2.67 (dd, *J* = 13.3, 7.5 Hz, 1H), 2.46 (dd, *J* = 13.3, 5.0 Hz, 1H), 1.40 (d, *J* = 7.2 Hz, 3H), 1.34 (s, 9H); ^13^C-NMR (100 MHz, CDCl_3_) (δ, ppm): 170.08, 155.76, 144.26, 129.55, 128.17, 127.01, 118.73, 80.88, 77.23, 67.49, 35.75, 29.71, 28.22, 19.36.

Preparation of aminothiol-containing amidonitriles **11** and **12**

(*R*)-1-(((*S*)-1-cyano-3-(methylthio)propyl)amino)-3-sulfanyl-1-oxopropan-2-aminium trifluoroacetate (**11**)


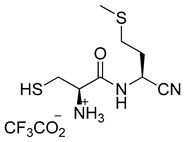


Nitrile **9** (30 mg, 0.050 mmol) was dissolved in DCM (2 mL), and *i*Pr_3_SiH (82 µL, 0.40 mmol) and TFA (77 µL, 1.00 mmol) were added. The mixture was stirred for 1 h and then it was extracted with D_2_O (1.5 mL). Finally, the aqueous layer was left under vacuum at room temperature to remove the remaining DCM, and the required product **11** was isolated as its TFA salt.

^1^H-NMR (500 MHz, D_2_O) (δ, ppm): 4.81–4.77 (m, 1H), 4.03 (t, *J* = 5.7 Hz, 1H), 2.96–2.81 (m, 2H), 2.53–2.36 (m, 2H), 2.13–1.95 (m, 2H), 1.90 (s, 3H); ^13^C-NMR (125 MHz, D_2_O) (δ, ppm): 167.79, 118.17, 54.19, 39.92, 30.13, 28.58, 24.52, 13.86.

(*R*)-1-(((*S*)-1-cyanoethyl)amino)-3-sulfanyl-1-oxopropan-2-aminium trifluoroacetate (**12**)


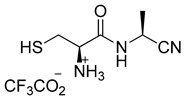


Nitrile **10** (30 mg, 0.058 mmol) was dissolved in DCM (2 mL), and *i*Pr_3_SiH (85 µL, 0.46 mmol) and TFA (89 µL, 1.16 mmol) were added. The mixture was stirred for 1 h and then it was extracted with D_2_O (1.5 mL). Finally, the aqueous layer was left under vacuum at room temperature to remove the remaining DCM, and the required product **12** was isolated as its TFA salt.

^1^H-NMR (500 MHz, D_2_O) (δ, ppm): 4.77 (q, *J* = 7.2 Hz, 1H), 4.23–4.03 (m, 1H), 3.14–2.88 (m, 2H), 1.52 (d, *J* = 7.3 Hz, 3H); ^13^C-NMR (125 MHz, D_2_O) (δ, ppm): 167.65, 119.41, 54.20, 36.78, 24.62, 16.92.

For more details, see Supplementary Files.

## 3. Results

As indicated in the Materials and Methods section, we have chosen to carry out non-prebiotic syntheses of our starting compounds, which allowed us to obtain homogeneous materials **11** and **12**. As we expected the formation of complex mixtures from these materials, this was a necessary compromise between authenticity and feasibility of the mixture analyses. On the primitive Earth, these compounds could have been obtained by one of the many prebiotic methods proposed for the formation of amide bonds [24].

When the salts of the activated derivatives **11** and **12** were left in aqueous solution (pH fixed at 6.5–7 by adding sodium bicarbonate), the solution became cloudy after a few hours, and a white precipitate was deposited after one day. For NMR studies, the mixtures were left in NMR tubes without further purification. For MALDI analysis, the precipitate was filtered, washed several times with water, and, finally, dried under vacuum.

### 3.1. Formation of Cysteine–Methionine Polymers

**Scheme 3:**
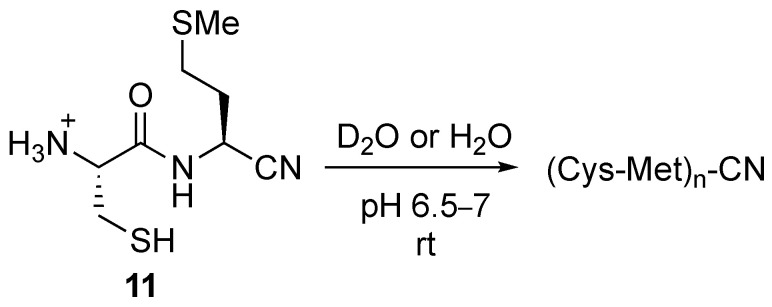
Polymerization of amidonitrile **11**.

The first test was run in deuterated water (D_2_O). After the deprotection of compound **9**, Cys-Met-CN **11** was extracted as a TFA salt in D_2_O. The obtained nitrile was found to be stable in acidic water (pH 1–2) for several days. The acidic solution was neutralized using sodium bicarbonate to reach a neutral pH (6.5–7). At this stage, we observed a turbid solution that, within a day, gave a white solid precipitate. This indicates the formation of polymeric materials based on cysteine and methionine amino acids (Scheme 3). 

**Figure 2:**
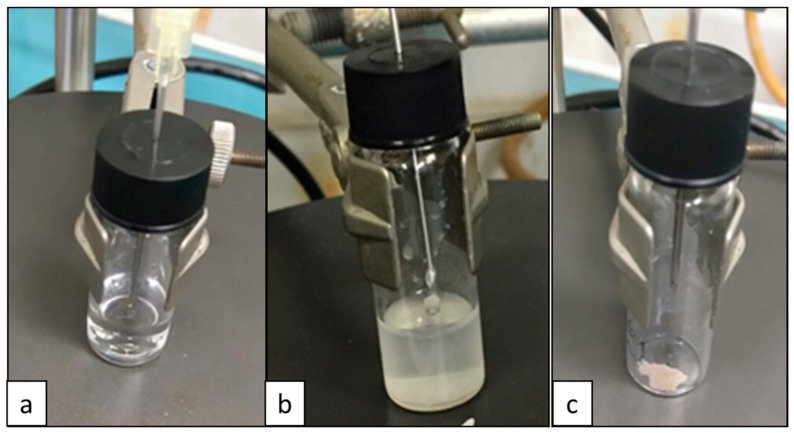
General observations during the polymerization process of nitriles **11** and **12** under argon. (**a**) Nitrile monomer dissolved in degassed water, (**b**) solution turning turbid after a few hours, (**c**) solid recovered after washing and drying.

The experiment was conducted again using degassed water H_2_O. After allowing the solution to remain undisturbed at room temperature overnight, a thin white solid layer was seen at the container’s base (Figure 2). This solid was extracted, dissolved in DMSO, and analyzed through MALDI spectrometry to discern the peptide mixture present.

**Figure 3:**
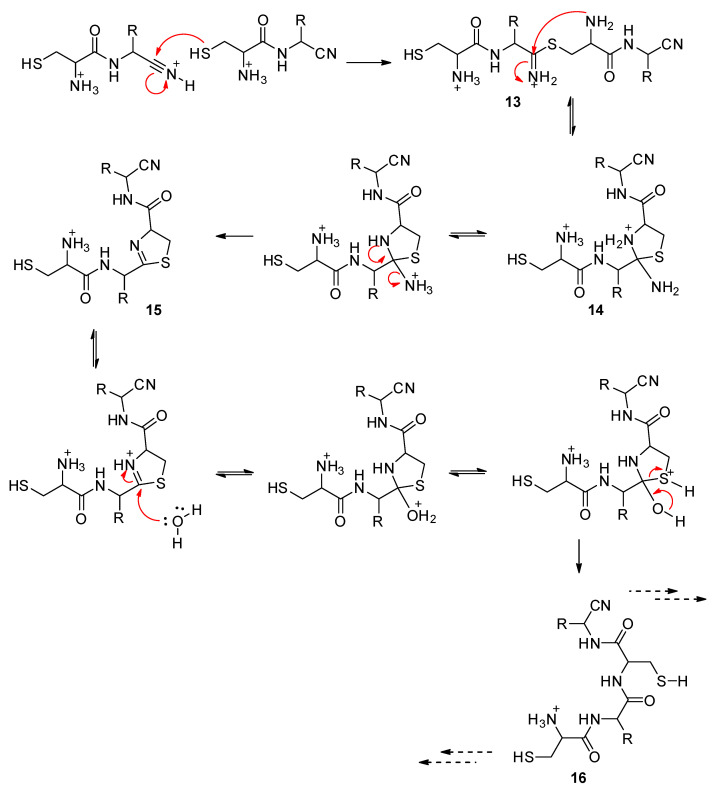
Proposed mechanism for the polymerization of nitriles **11** and **12**.

The polymerization process starts with the formation of thiazoline rings (Figure 3) [23]. A nucleophilic attack of the thiol of one unit on the nitrile group of a second unit is the starting point. Then, the amine adds onto the obtained iminium double bond in **13** to form a transient thiazolidine **14**, which, upon NH_3_ loss, gives the more stable thiazoline cycle **15**. This thiazoline is then slowly hydrolyzed to give dipeptide **16**. The formed dimer **16** still has both active sides (a thiol and a nitrile) and it can continue the polymerization process to reach higher-length peptides, which indeed have been observed in mass spectrometry analysis.

**Figure 4:**
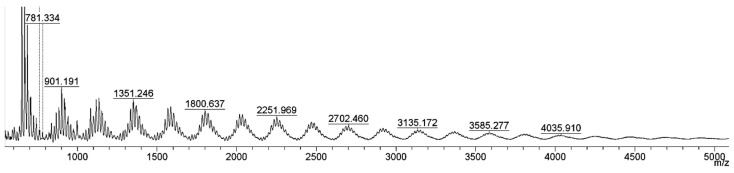
MALDI MS spectrum of poly(Cys-Met), positive ionization.

The MALDI mass spectrometry analysis (Figure 4) revealed multiple polypeptide chains of varying lengths up to 25 units (which corresponds to a 50 amino acid sequence). The spectrum that was obtained did not present the anticipated peptides as individual masses (one mass for each (Cys-Met)_n_). Instead, a collection of masses was observed for each given peptide length. These masses were attributed to the presence of a varied number of hydrolyzed and non-hydrolyzed thiazoline rings in each chain.

**Figure 5:**
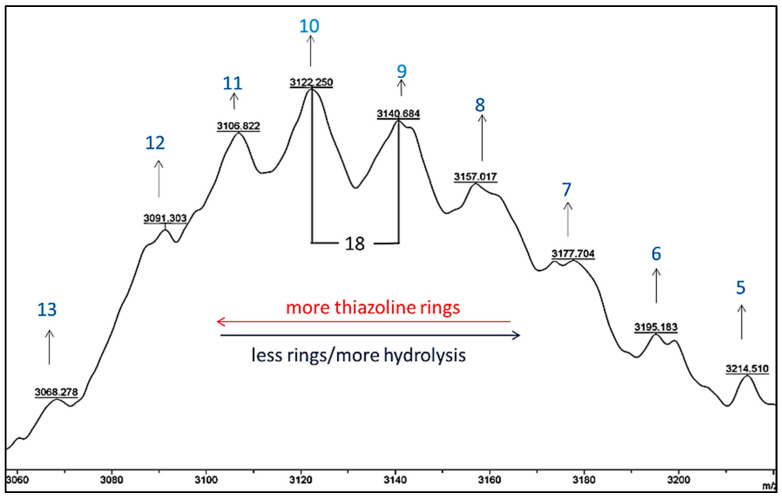
Peaks corresponding to (Cys-Met)_14_-CN on MALDI; numbers in blue correspond to the count of thiazoline rings.

As an example, the multiple masses detected for (Cys-Met)_14_-CN are shown in Figure 5. The first (and lowest) mass detected in this zone was 3068.278, which is compatible with the presence of thirteen thiazoline rings, the highest number of thiazoline rings possible for fourteen Cys-Met units. The following mass, 3091.303, corresponds to one amide (e.g., one hydrolyzed thiazoline) and twelve thiazoline rings. The mass, 3106.822, stands for eleven thiazoline rings, and so on. With less rings, higher masses are expected; the difference between two peaks is 18, the mass of a water molecule, which is required for hydrolysis of one thiazoline ring.

**Figure 6:**
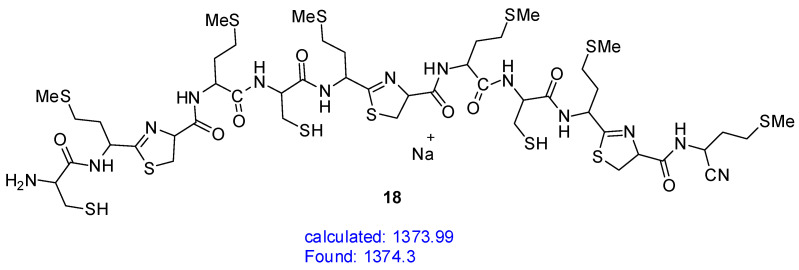
A possible structure for M = 1374.3.

As an example, the peptide structure **18,** which consists of six Cys-Met units, contains three thiazoline rings, and ends with a nitrile, is presented in Figure 6.

Most observed masses belonged to peptides ending with a nitrile function. However, we also observed some chains ending with a carboxylic acid group (resulting from nitrile hydrolysis). Some other examples of masses observed from MALDI experiments are given in Table 1.

**Figure 7:**
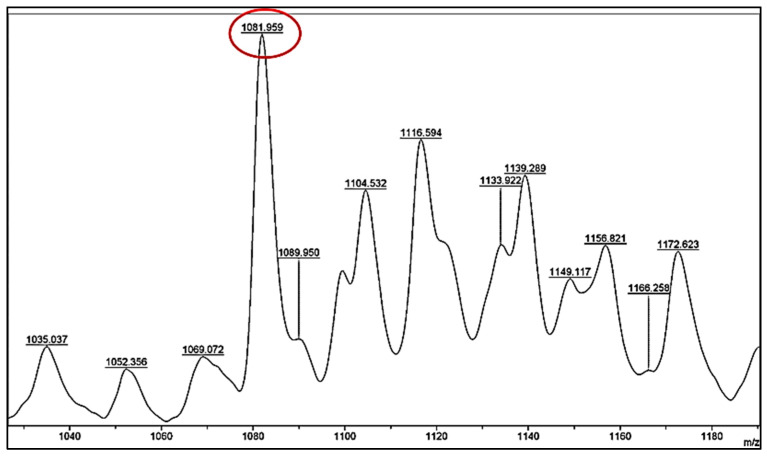
An odd peak within other mass peaks, which corresponds to a macrocyclic structure.

In many cases, if we focus on a specific region in the MALDI spectrum, we notice an odd peak that is not consistent with the rest of the distribution. An example of such a unique peak is presented in Figure 7.

**Figure 8:**
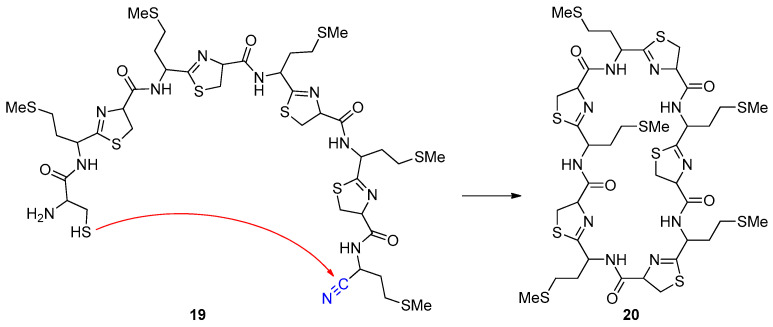
Cyclization of fully thiazoline-linked oligomer **19** into **20**.

At any degree of polymerization, the obtained polymer contains the two active functions: a nitrile group and an aminothiol. Thus, the molecule has two pathways to choose from: either to react with another monomer in order to give a longer peptide chain, or to perform a cyclization reaction to give a macrocycle. For example, (Cys-Met)_5_-CN **19** (Figure 8) can undertake further reaction to give a longer linear peptide chain, or it may cyclize if the thiol function at the opposite end of the chain performs an intramolecular reaction with the nitrile. Such a process eventually yields the macrocycle **20**, containing five thiazoline rings, which agrees with the observed mass of 1081.959 (Figure 7). The observed structures of cyclic poly(Cys-Met) are presented in Table 2.

**Figure 9:**
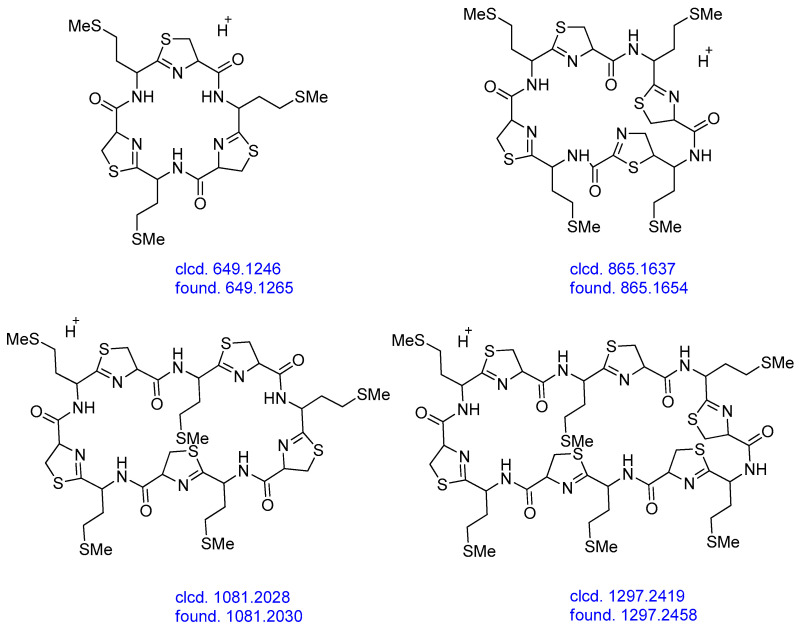
Exact masses of detected cyclic structures of poly(Cys-Met).

Masses presented in Table 2 are average masses. To confirm the proposed structures, we also performed high-resolution mass spectrometry (HRMS-SEI). As shown in Figure 9, the found exact masses agree with the proposed thiazoline-containing cyclopeptides cyclo(CysMet)_n_ for n between 3 and 6. Unfortunately, the quantities of cyclo(CysMet)_7_ and cyclo(CysMet)_8_ in the mixture of polymers were not sufficient to allow the measurement of their exact masses.

In the case of the observed macrocycles, none of the thiazoline rings underwent hydrolysis. It is probable that the bent geometry of the chain containing thiazoline rings favors the reaction between its two active ends. Precipitation of these molecules then prevents any hydrolysis from occurring.

### 3.2. Formation of Cysteine–Alanine Polymers

**Scheme 4:**
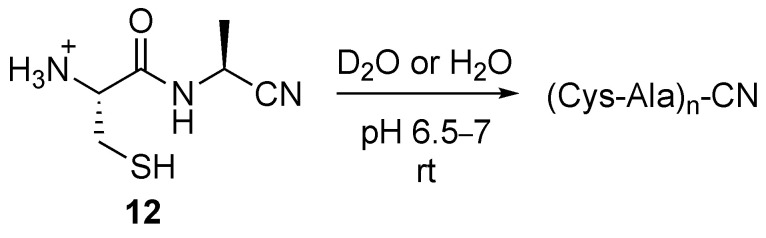
Polymerization of amidonitrile **12**.

The behaviour of Cys-Ala-CN **12** was very similar to the behaviour of **11**. It was stable in acidic water, but a precipitate formed when the pH was raised to 6.5–7. The solution started to become turbid, then a white solid slowly precipitated out of the solution, indicating the formation of polymeric materials (Scheme 4).

**Figure 10:**
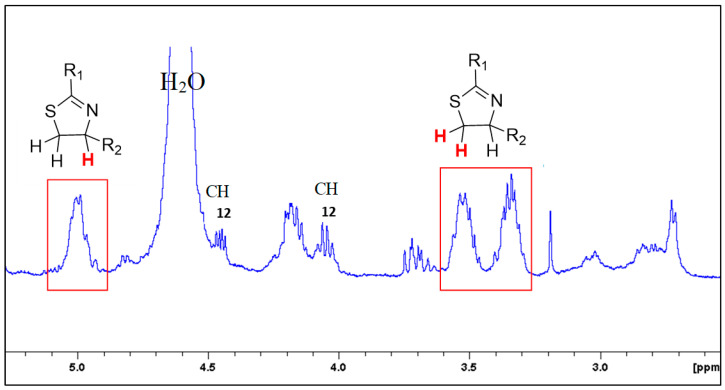
Observation of thiazoline rings of poly(Cys-Ala) in ^1^H-NMR.

As shown in Figure 10, thiazoline rings were characterized by ^1^H-NMR spectroscopy thanks to the appearance of new signals around 5 ppm (CH of thiazolines) and in the region from 3.3 to 3.6 ppm (CH_2_ of thiazolines).

**Figure 11:**
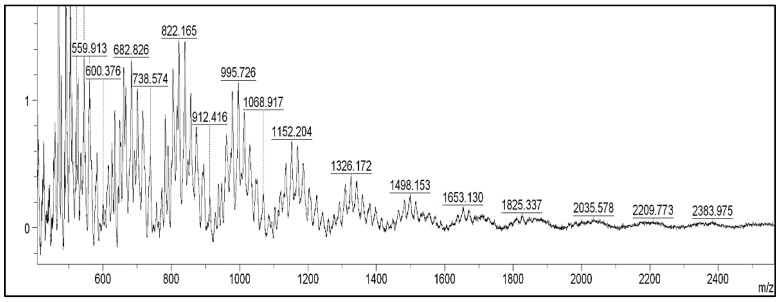
MALDI MS spectrum of poly(Cys-Ala).

The experiment was repeated in degassed H_2_O in order to perform mass spectrometry. A solution of **12** was neutralized by sodium bicarbonate, and the solution was left unstirred overnight under inert atmosphere to give a white powder at the bottom of the reaction container. Then, the solid was recovered and subjected to MALDI-TOF analysis (Figure 11).

**Figure 12:**
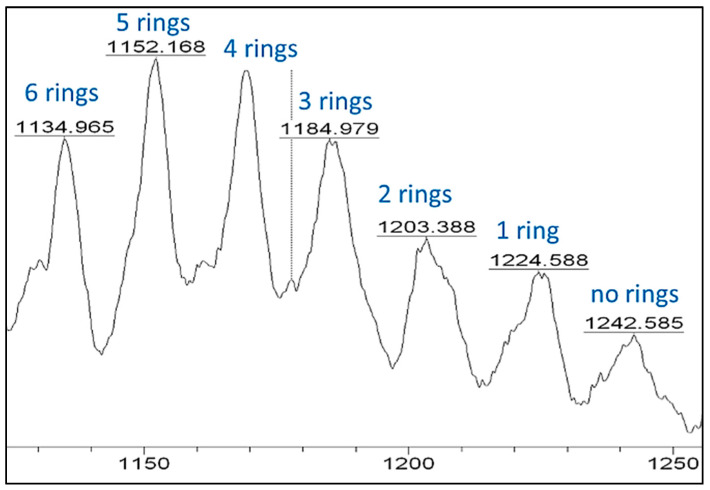
Peaks corresponding to (Cys-Ala)_7_-CN on MALDI spectrometry, [M + Na]^+^.

Taking as an example the region of masses shown in Figure 12, we can observe seven single masses, which correspond to seven Cys-Ala units that end with a nitrile. The highest number of possible thiazoline rings in the case of (Cys-Ala)_7_-CN is six rings, and this is reflected in the calculated mass of 1134.4598 (observed 1134.965). The adjacent peak, 1152.168, contains one hydrolyzed thiazoline ring, and five rings are still present.

**Figure 13:**
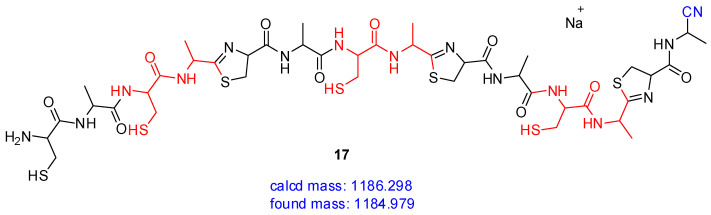
A possible structure for [M + Na]^+^ = 1184.979.

It is impossible to discern the exact location of the thiazoline rings in the chain. For instance, the peak of 1184.979, observed in (Cys-Ala)_7_-CN, is likely due to a mixture of oligomers with three hydrolyzed and three remaining thiazoline rings. All of these structures are heptamers of Cys-Ala, meaning that they consist of a chain of fourteen amino acids **17** (Figure 13).

Some examples of the found linear polypeptides that contain thiazoline rings are shown in Table 3.

## 4. Discussion

What we have tried to prove here is that starting from relatively simple molecules, complex mixtures can be obtained [25]. Thus, from the aminothiol-containing amidonitrile **11** (Cys-Met-CN) or **12** (Cys-Ala-CN), an array of compounds is observed after a few hours. Polymers where the Cys-AA repeat units are linked through either a thiazoline ring or an amide bond were detected, with chains bearing up to 50 amino acid residues (25 repetitions of Cys-AA). Macrocycles with up to 16 amino acid residues were also formed from fully thiazoline-linked polymers. The 3D structures of these macromolecules are therefore very diverse. The importance of thiazoles (the oxidized equivalents of thiazolidines) must be emphasized [26,27]. For example, thiamine pyrophosphate, containing a key thiazole ring and present in all living things, cooperates with pyruvate synthase to catalyze one of the oldest anabolic methods of CO_2_ fixation, the Wood–Ljungdahl pathway [28,29]. The presence of thiazoles in the primitive environment may have facilitated the rise of a proto-metabolism.

For this spontaneous diversification to happen, the starting molecules must be functionalized and even multi-functionalized. Our starting molecules contain an amide function, which is poorly reactive, a nitrile, an amine, and a thiol. Indeed, nitriles are not so reactive; for instance, their hydrolysis is pretty slow [30], and their reaction with alcohols (the so-called Pinner reaction) [31] requires a strong acid catalyst. Amines are nucleophilic. Unfortunately, they are protonated in water, even at a moderately basic pH, which makes their reactivity dependent on the presence of scarce amounts of their non-protonated forms. Thiols do not face this problem. They are more reactive when deprotonated (pH > 7–8), but remain nucleophilic even in their neutral form.

Of the four functions present in our starting molecules, it is indeed the thiol function that plays the leading role. It is sufficiently nucleophilic to attack the nitrile group of a second molecule, even if this nitrile is relatively weakly electrophilic. Without this thiol, nothing would happen (except probably the slow hydrolysis of the nitrile). It is only after the action of the thiol that the amine intervenes. This amine is, of course, protonated most of the time, but as soon as it is deprotonated, its free form attacks the intermediate C=N double bond. This is a rapid attack because it corresponds to the closing of a five-membered cycle. The attack of the thiol is a prerequisite [32].

We believe that at the origin of life, thiols, which are easy to obtain and which are reactive in any conditions (except perhaps at a very low temperature), participated in the creation of the necessary molecular diversity in which life picked up the molecules that were essential to its development.

To go towards even more diversity, it would of course be appropriate to mix, for example, several amidonitriles. However, this would create such complex mixtures that they could no longer be analyzed by mass spectrometry (at least not by the techniques at our disposal). A whole world of molecules can emerge from actually very simple starting compounds; a world of linear chains and cycles, big and small, and not just thiazolines, of course [33,34,35]. Chemistry before life may have been a “prebiotic mess” from which emerged the chemistry of life today; as complex as this may seem to us, it is perhaps only a small extract.

## Figures and Tables

**Table 1:** Some representative products obtained after polymerization of amidonitrile **11**.

No. of Monomers	No. of Thiazolines	Calculated Molecular wt. [M + Na]^+^	Observed Average Mass [M + Na]^+^
(Cys-Met)_13_-Cys-Met-CN	13	3068.5522	3068.278
(Cys-Met)_13_-Cys-Met-CN	10	3122.5981	3122.250
(Cys-Met)_13_-Cys-Met-CN	9	3140.6134	3140.684
(Cys-Met)_14_-Cys-Met-CN	9	3374.9524	3375.55
(Cys-Met)_15_-Cys-Met-CN	11	3573.2608	3573.852
(Cys-Met)_15_-Cys-Met-CN	8	3627.3067	3626.564

**Table 2:** Detected macrocycle masses observed in the mixture of polymers obtained from **11**.

No. of Monomers	No. of Thiazolines	Chemical Formula	Calculated Molecular wt. [M + H]^+^	Observed Average Mass [M + H]^+^
Cyclo(Cys-Met)_3_	3	C_24_H_37_N_6_O_3_S_6_^+^	649.978	649.074
Cyclo(Cys-Met)_4_	4	C_32_H_49_N_8_O_4_S_8_^+^	866.3027	865.745
Cyclo(Cys-Met)_5_	5	C_40_H_61_N_10_O_5_S_10_^+^	1082.6264	1081.959
Cyclo(Cys-Met)_6_	6	C_48_H_73_N_12_O_6_S_12_^+^	1298.9501	1298.332
Cyclo(Cys-Met)_7_	7	C_56_H_85_N_14_O_7_S_14_^+^	1515.2738	1515.213
Cyclo(Cys-Met)_8_	8	C_64_H_97_N_16_O_8_S_16_^+^	1731.5975	1731.996

**Table 3:** Representative examples of (Cys-Ala)_n_ structures.

No. of Monomers	No. of Thiazolines	Calculated Molecular wt. [M + Na]^+^	Observed Average Mass [M + Na]^+^
(Cys-Ala)_5_-Cys-Ala-CN	5	977.2534	978.267
(Cys-Ala)_5_-Cys-Ala-CN	4	995.2687	995.765
(Cys-Ala)_5_-Cys-Ala-CN	3	1013.284	1013.111
(Cys-Ala)_6_-Cys-Ala-CN	6	1133.4589	1134.965
(Cys-Ala)_6_-Cys-Ala-CN	5	1151.4742	1152.168

## Data Availability

Not applicable.

## Supplementary Materials

The following supporting information can be downloaded at: https://www.mdpi.com/article/10.3390/life13040983/s1, supporting information containing detailed experimental part, ^1^H and ^13^C NMR spectra and MALDI spectra.
