# Supplementary material for: Straightforward Creation of Possibly Prebiotic Complex Mixtures of Thiol-Rich Peptides
Source: Life (Basel). 2023 Apr 10;13(4):983. doi: 10.3390/life13040983 (PMC10145665; doi:10.3390/life13040983)
Supplement: Supplementary file 1 [file life-13-00983-s001.zip › life-2341234-supplementary.pdf]

**Supplementary material for**

# Straightforward Creation of Possibly Prebiotic Complex Mixtures of Thiol-Rich Peptides

**Ibrahim Shalayel [1], Naoual Leqraa [2], Véronique Blandin [2] and Yannick Vallée [2,*]**

[1] Université Grenoble Alpes, TIMC-IMAG, CNRS, F-38000 Grenoble
[2] Université Grenoble Alpes, DCM, CNRS, F-38000 Grenoble
*Correspondence: yannick.vallee@univ-grenoble-alpes.fr

**Table of content**

**General experimental methods** ........ 2
**Copies of $^1$H NMR and $^{13}$C NMR spectroscopy** ........ 3
**MALDI MS Spectrum for poly(Cys-Met)** ........ 11
**Synthesis and analysis of the prepared molecules** ........ 12

General experimental methods

Standard handling techniques under an inert atmosphere were used for reagents sensitive to air or moisture. The reactions were monitored by thin layer chromatography (TLC) using commercial aluminum-backed silica gel plates (Merck, Kieselgel 60 F254). TLC spots were viewed under ultraviolet light, by heating the plate after treatment with Phosphomolybdic acid (PMA) solution or by heating the plate after treatment with Ninhydrin solution (1.5 g/100 mL *n*-butanol and 3 mL acetic acid). Product purifications by gravity column chromatography were performed using Macherey-Nagel silica gel 60 (70−230 mesh).

$^{1}$H-NMR and $^{13}$C-NMR spectra were recorded on a Bruker Avance 500 (1H: 500 MHz, 13C: 125 MHz) and 400 (1H: 400 MHz, 13C: 100 MHz) spectrometers.

Chemical shifts for $^{1}$H spectra are values from tetramethylsilane in $CDCl_3$ (δ 0.00 ppm) or relative to $H_2O$ peak in $D_2O$ (calibrated at 4.7 ppm). Chemical shifts for $^{13}$C spectra are values from $CDCl_3$ (δ 77.16 ppm).

$^{1}$H-NMR spectra are reported as following: chemical shift (ppm), multiplicity (s: singlet; d: doublet; dd: doublet of doublets; t: triplet; m: multiplet), coupling constants (Hz) and integration. Proton and carbon signal assignments were established using COSY, HSQC, and HMBC experiments.

High-resolution mass spectra (HRMS) were recorded on a Waters G2-S Q-TOF mass spectrometer and LTQ Orbitrap XL (Thermo Scientific). For MALDI analysis, DMSO was used as solvent to dissolve the obtained polymers and DHB (2,5-Dihydroxybenzoic acid) was used as matrix.

## Copies of $^{1}H$ NMR and $^{13}C$ NMR spectroscopy

## $^{1}H$ NMR and $^{13}C$ NMR spectroscopy of **3**

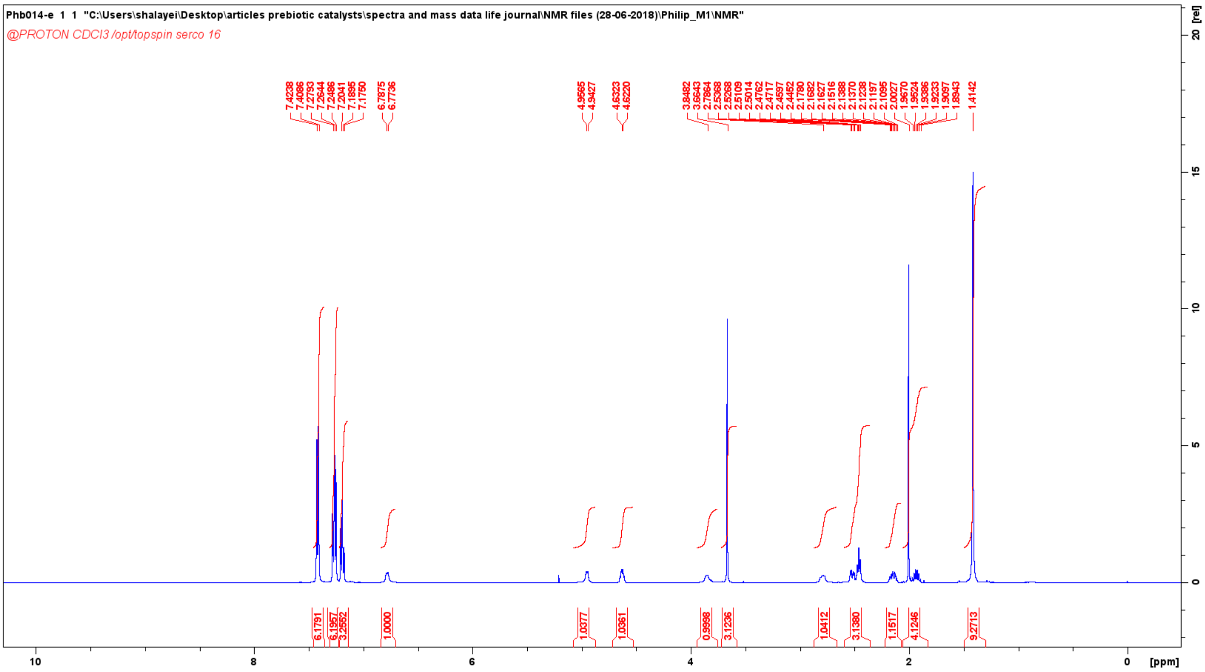


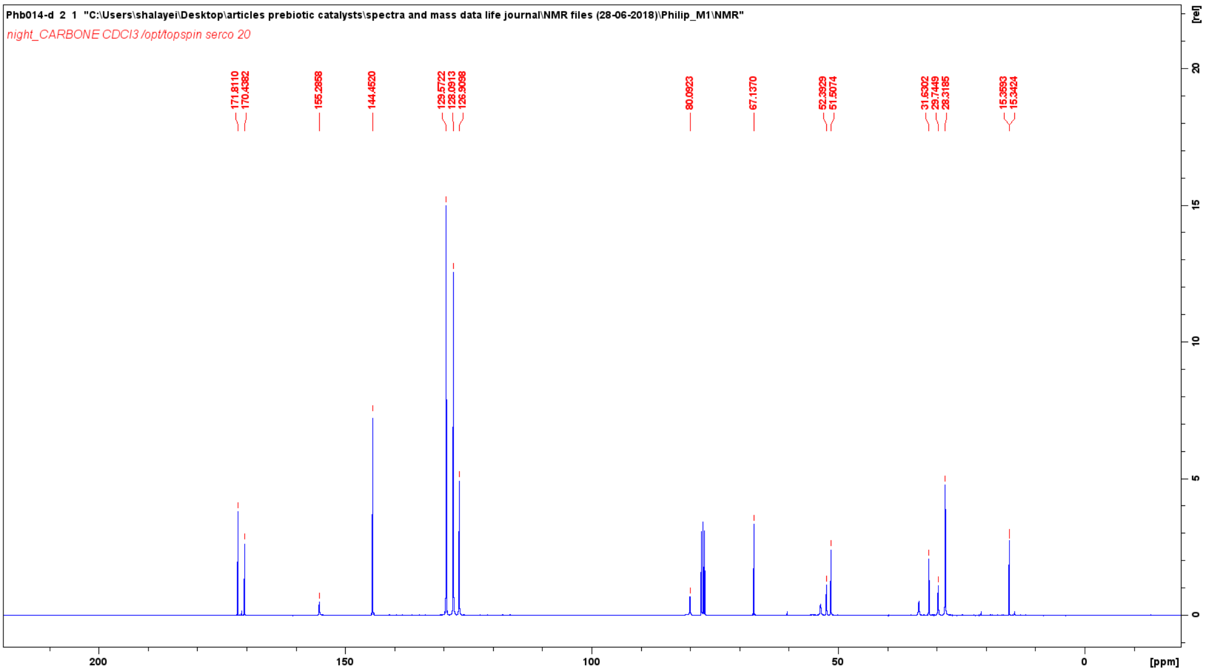

$^{1}$H NMR and DEPTQ spectroscopy of **4**

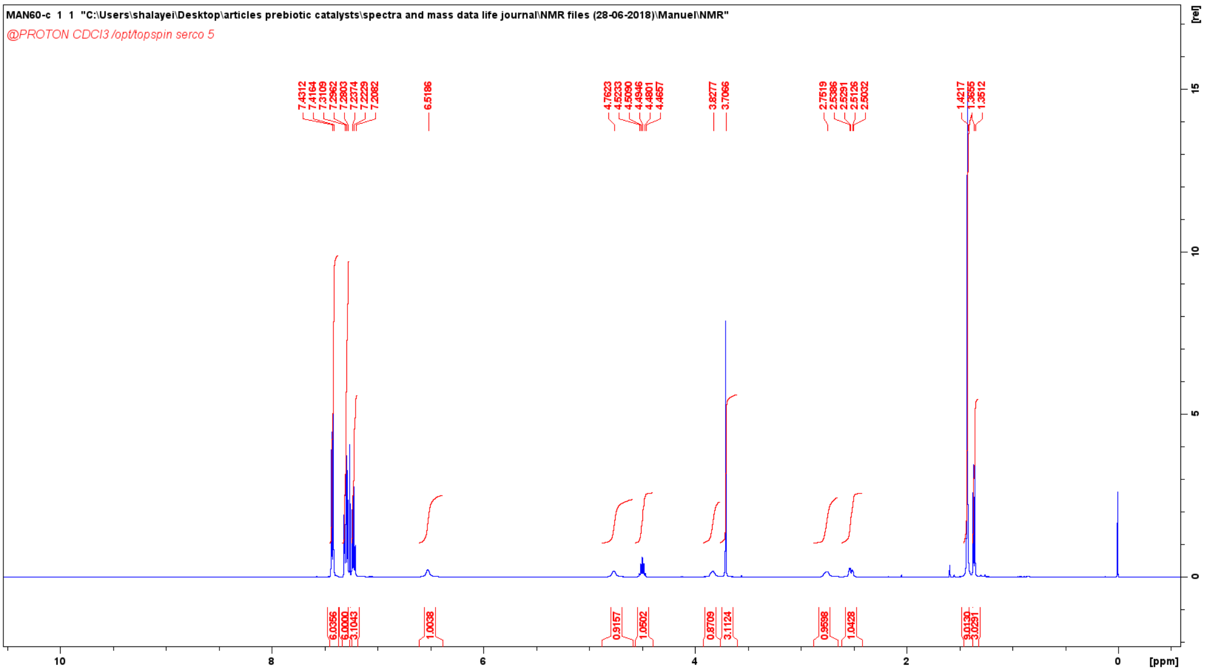


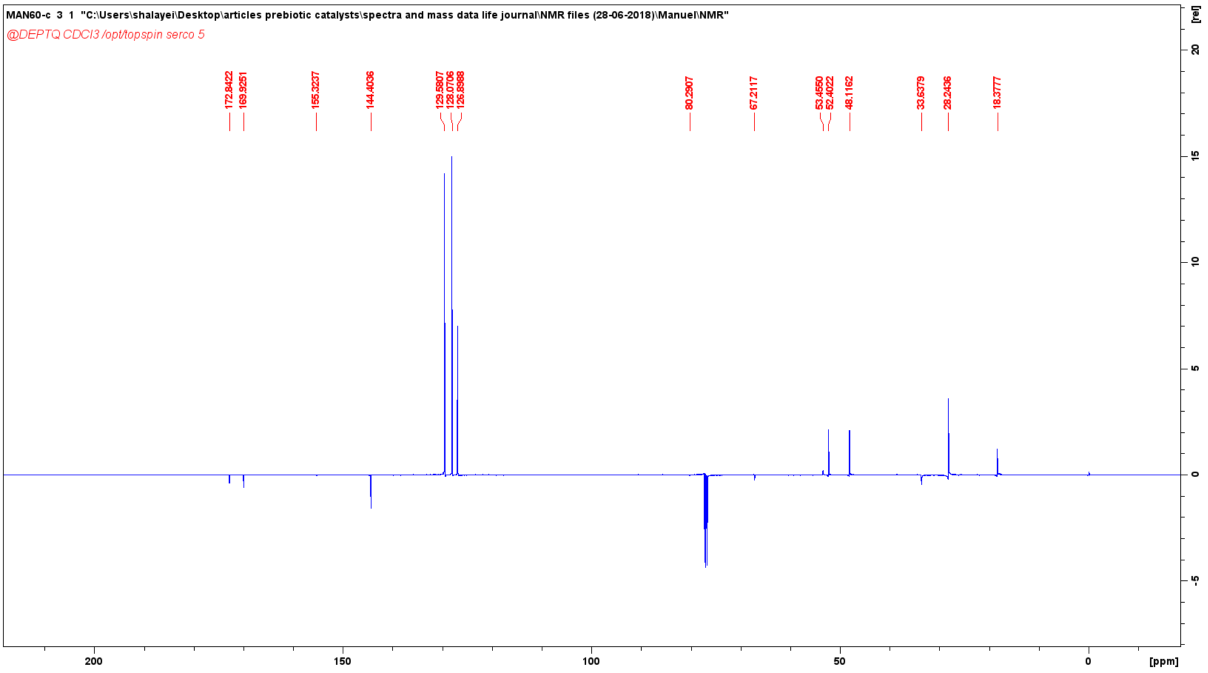

$^{1}$H NMR and $^{13}$C NMR spectroscopy of **5**

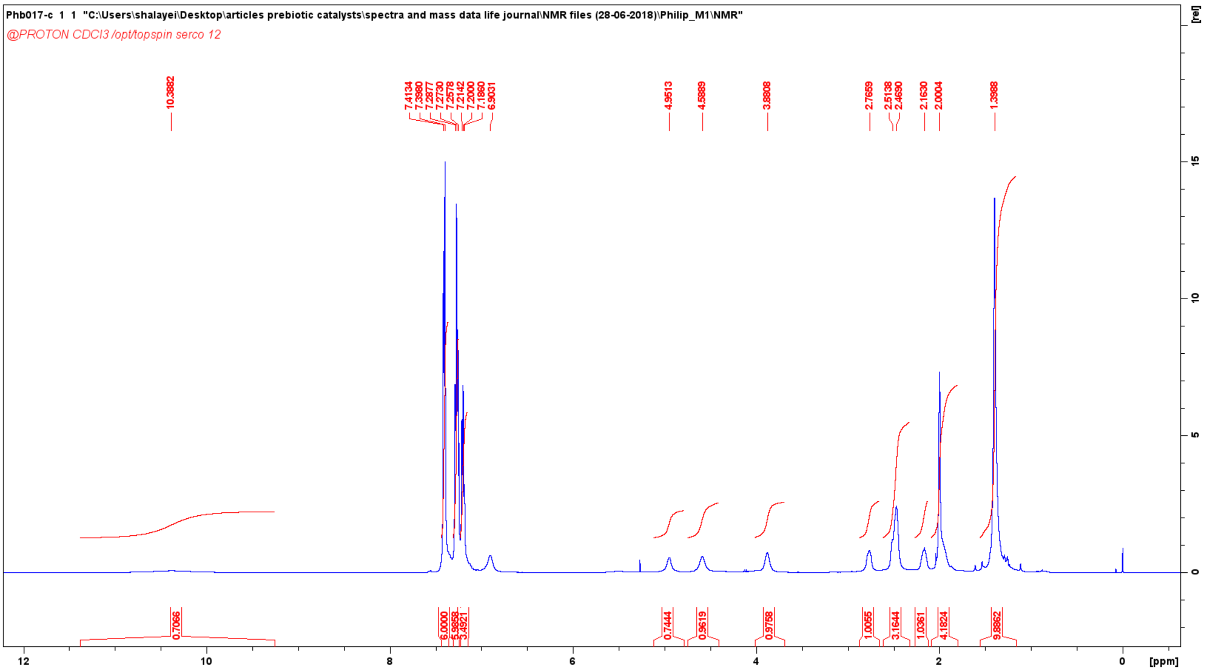


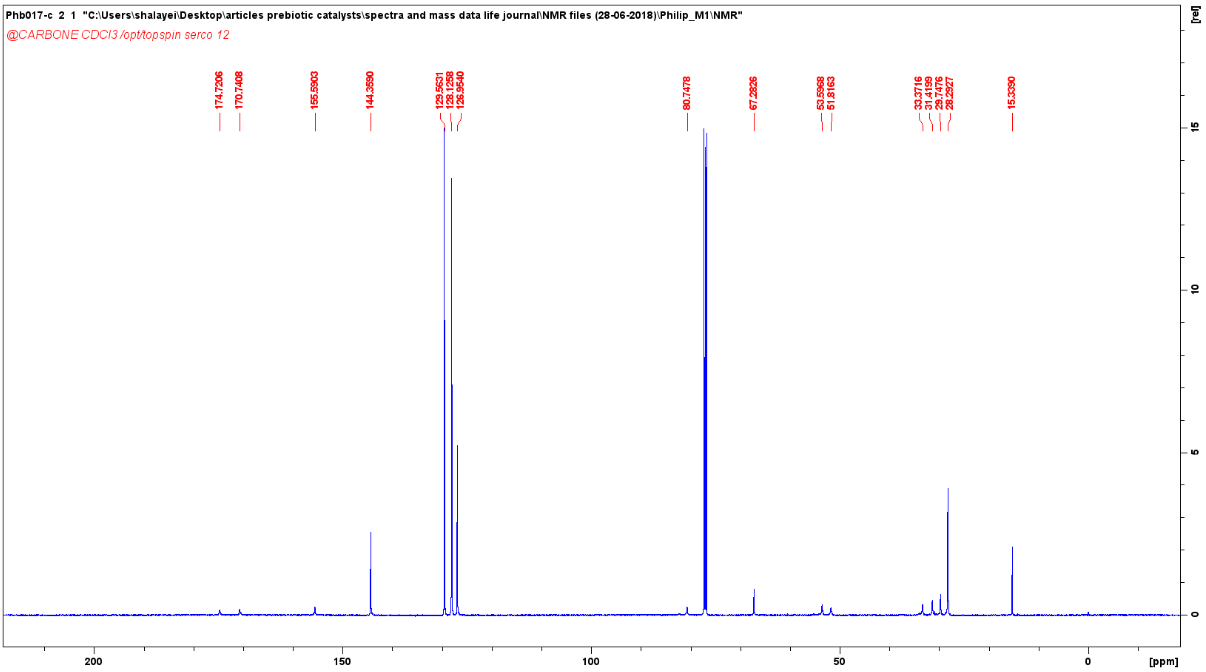

$^{1}$H NMR and $^{13}$C NMR spectroscopy of **7**

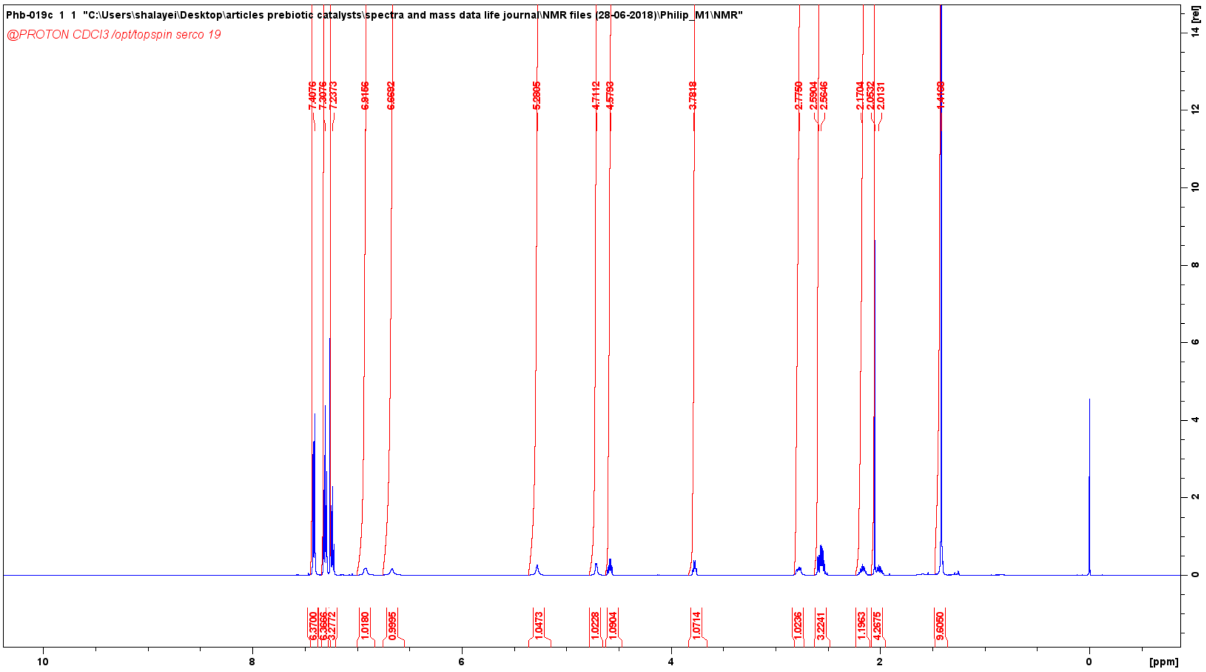


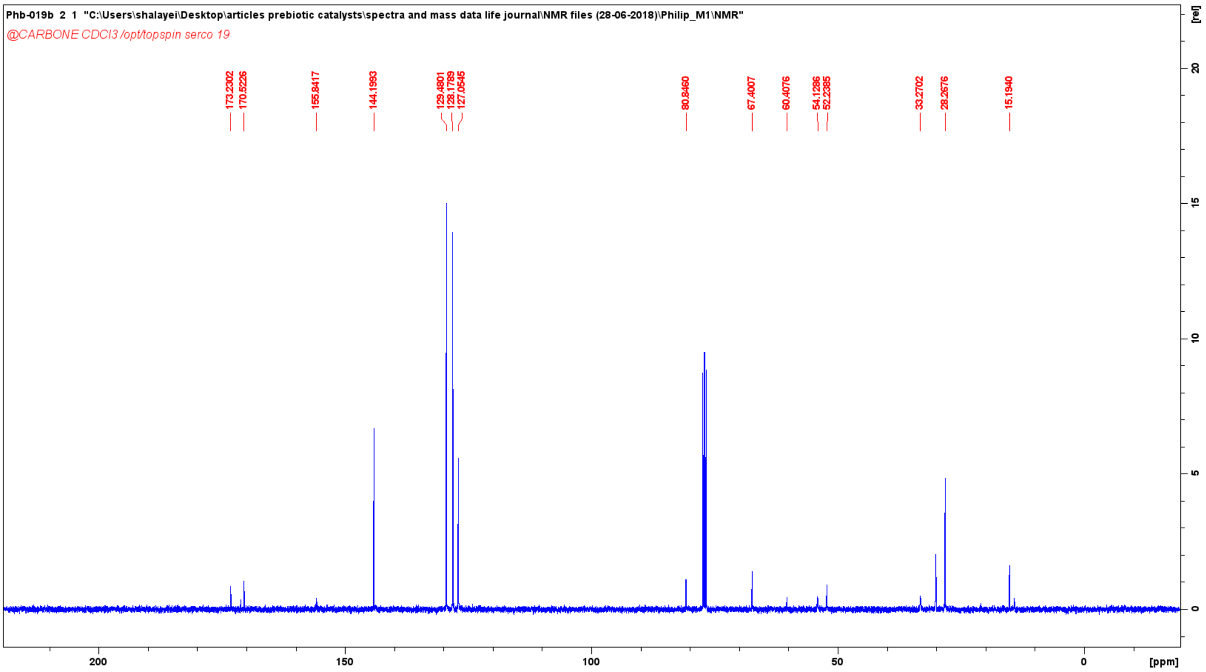

$^{1}$H NMR and DEPTQ spectroscopy of **8**

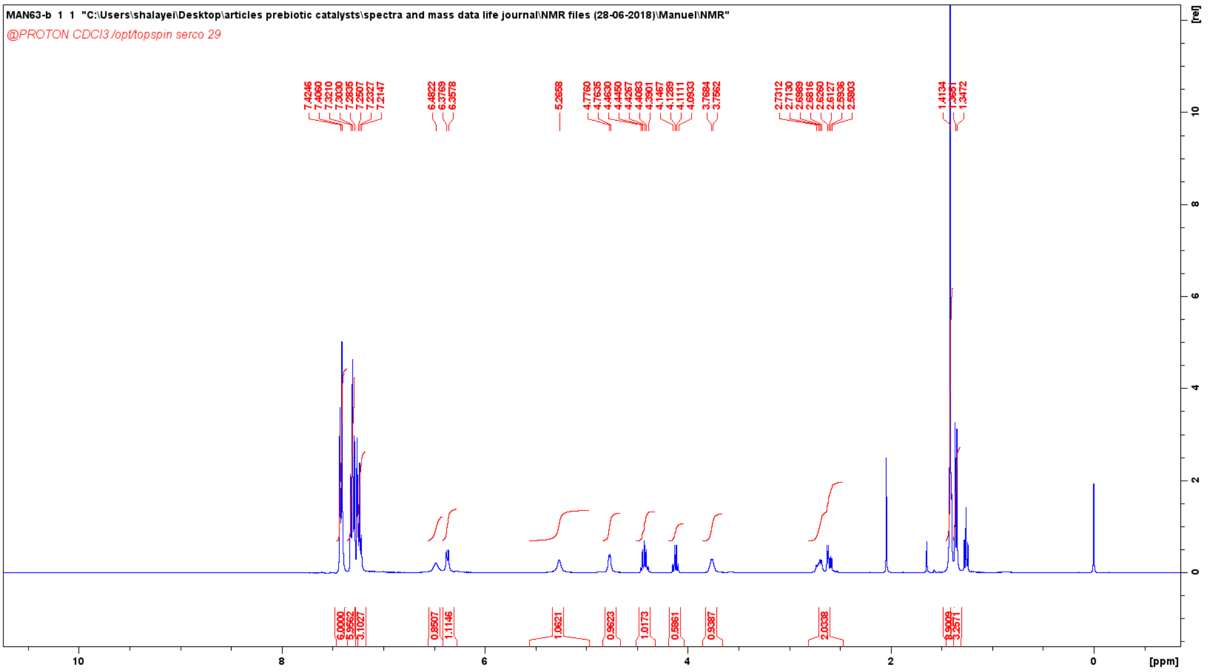


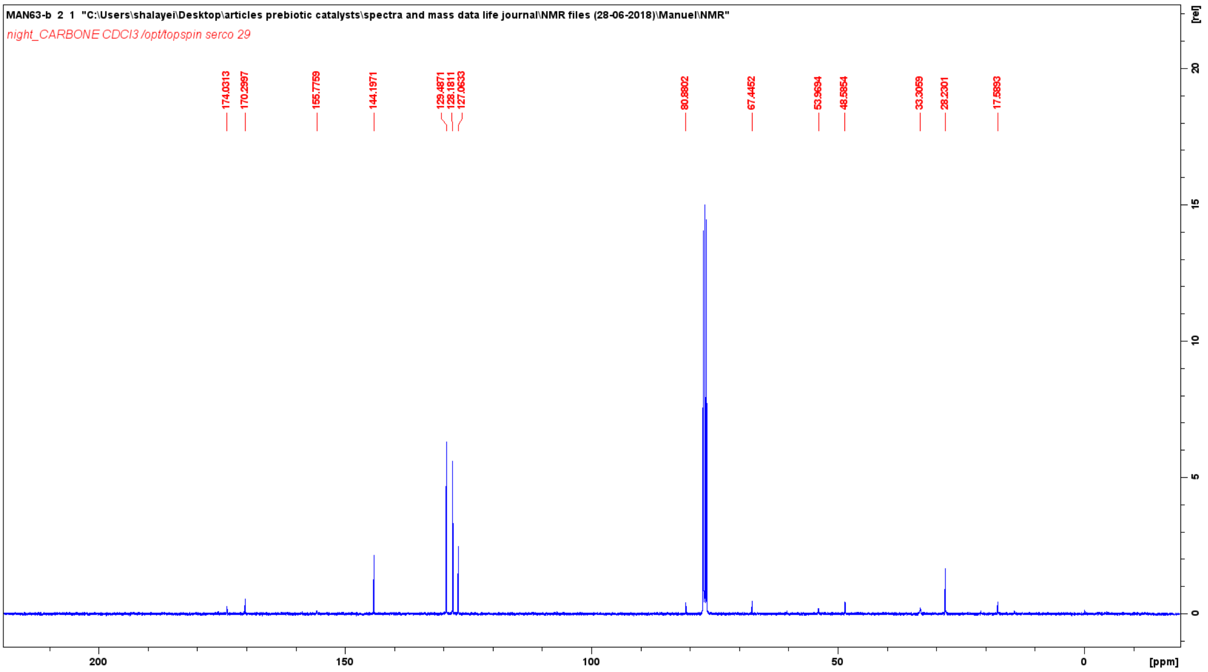

$^1H$ NMR and DEPTQ spectroscopy of **9**

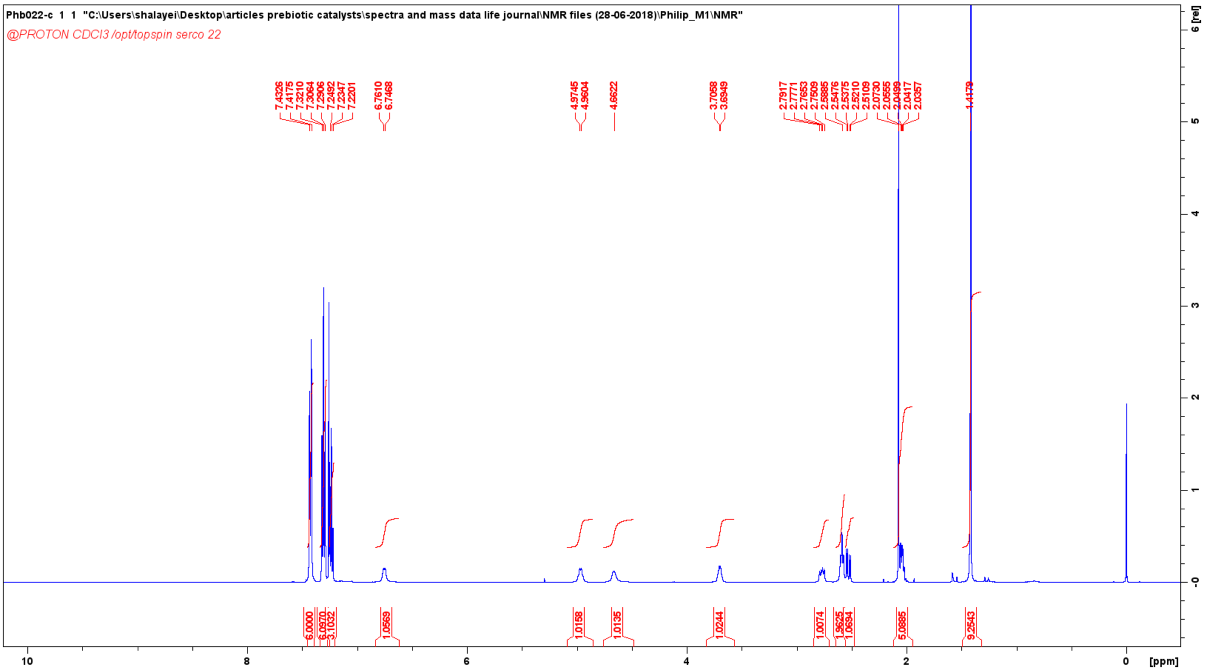

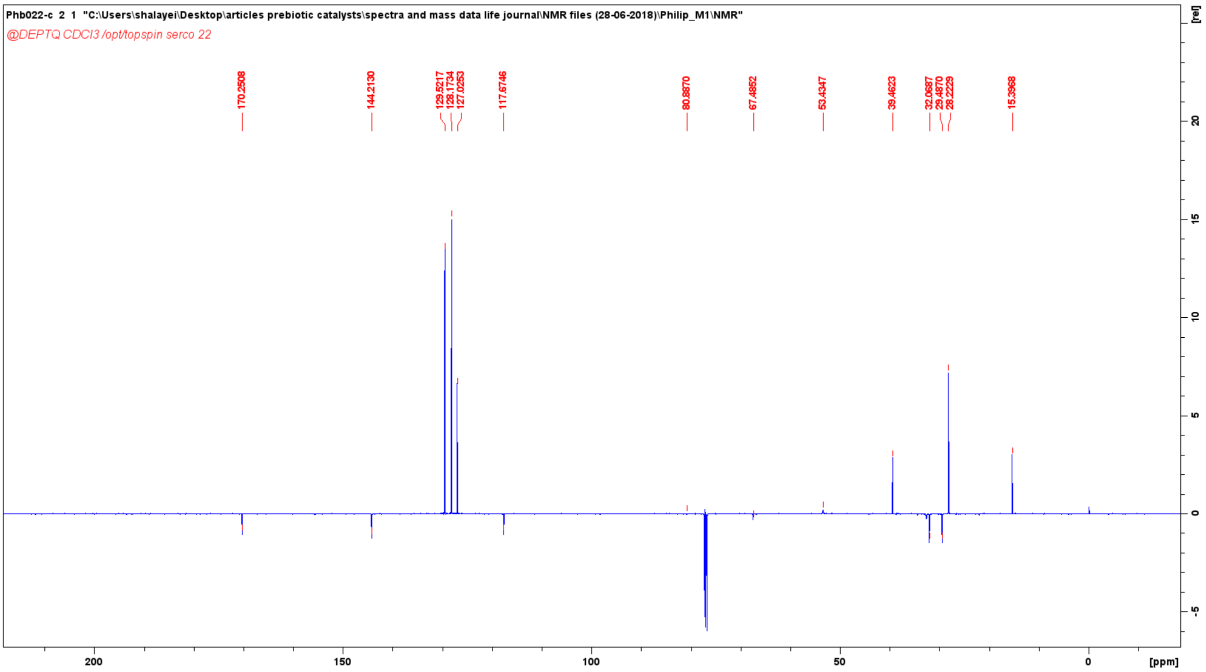

$^{1}H$ NMR and $^{13}C$ NMR spectroscopy of **10**

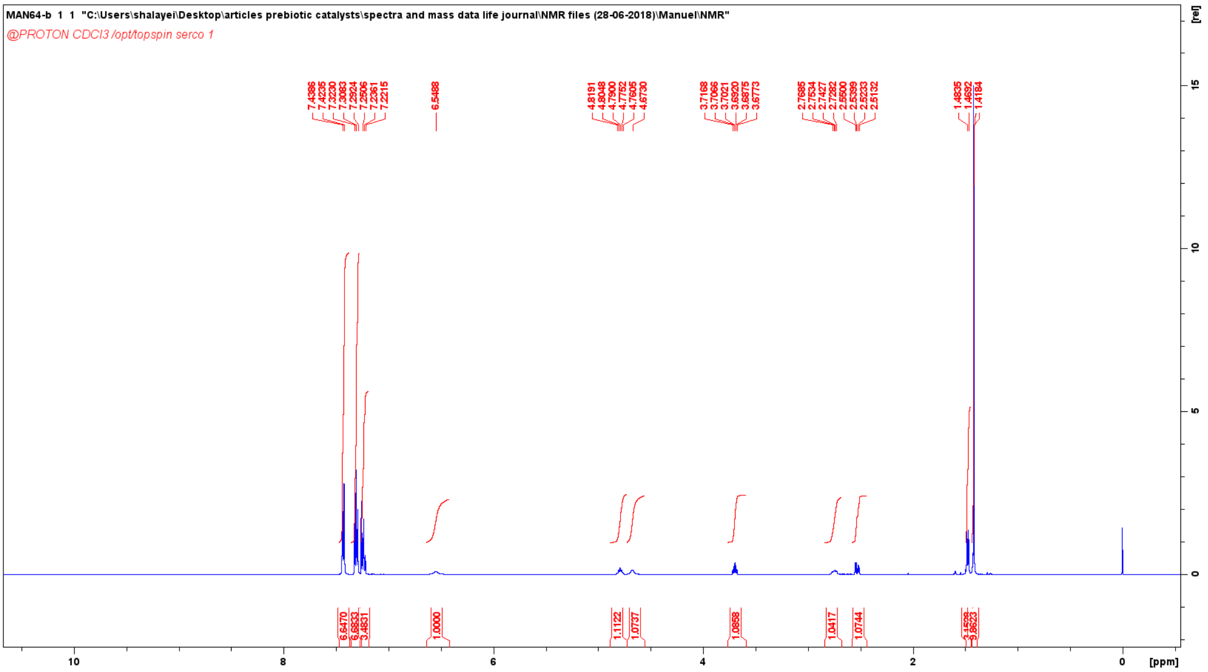


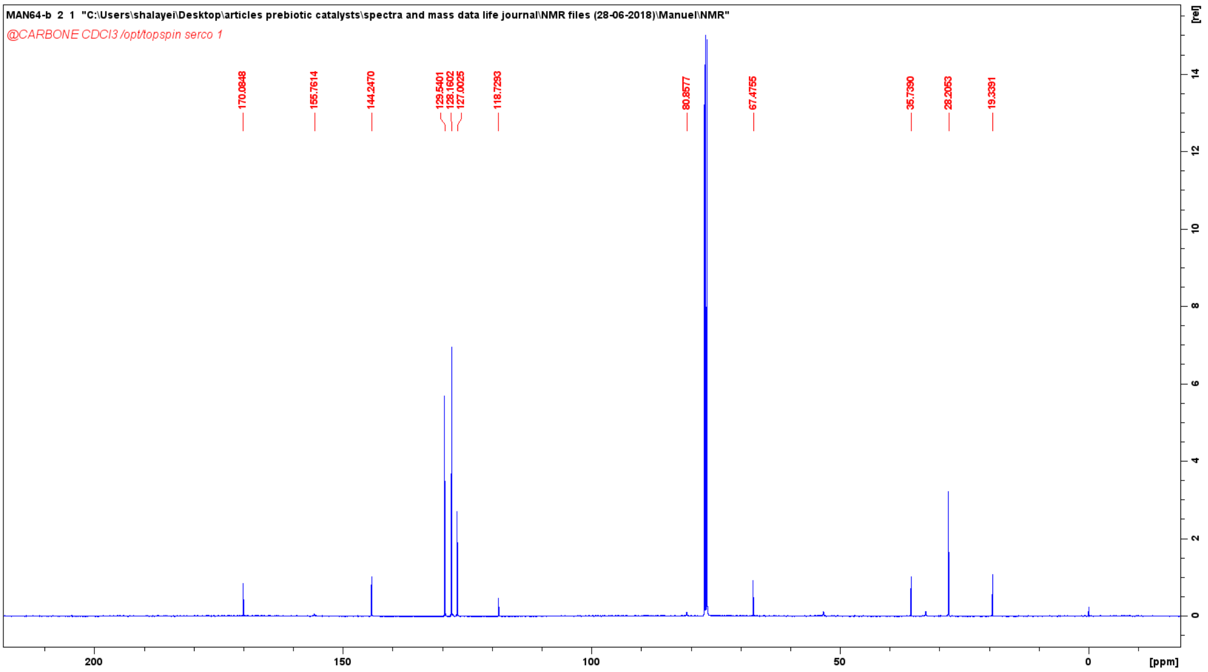

$^{1}H$ NMR and $^{13}C$ NMR spectroscopy of **11** (TFA salt)

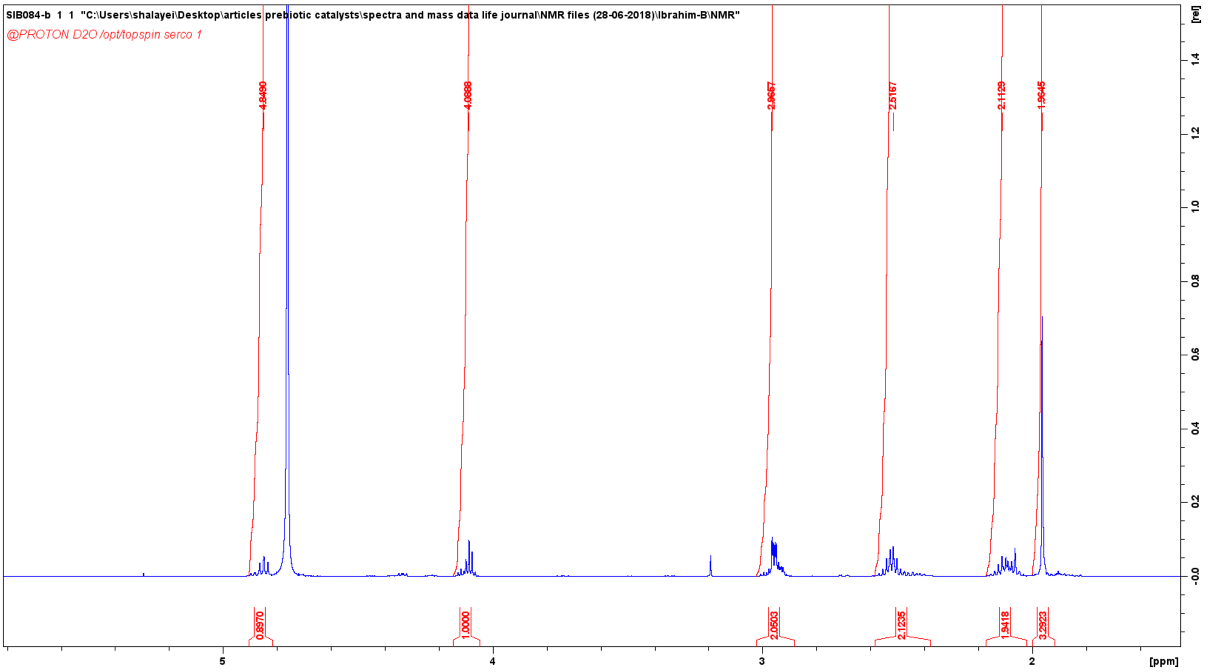


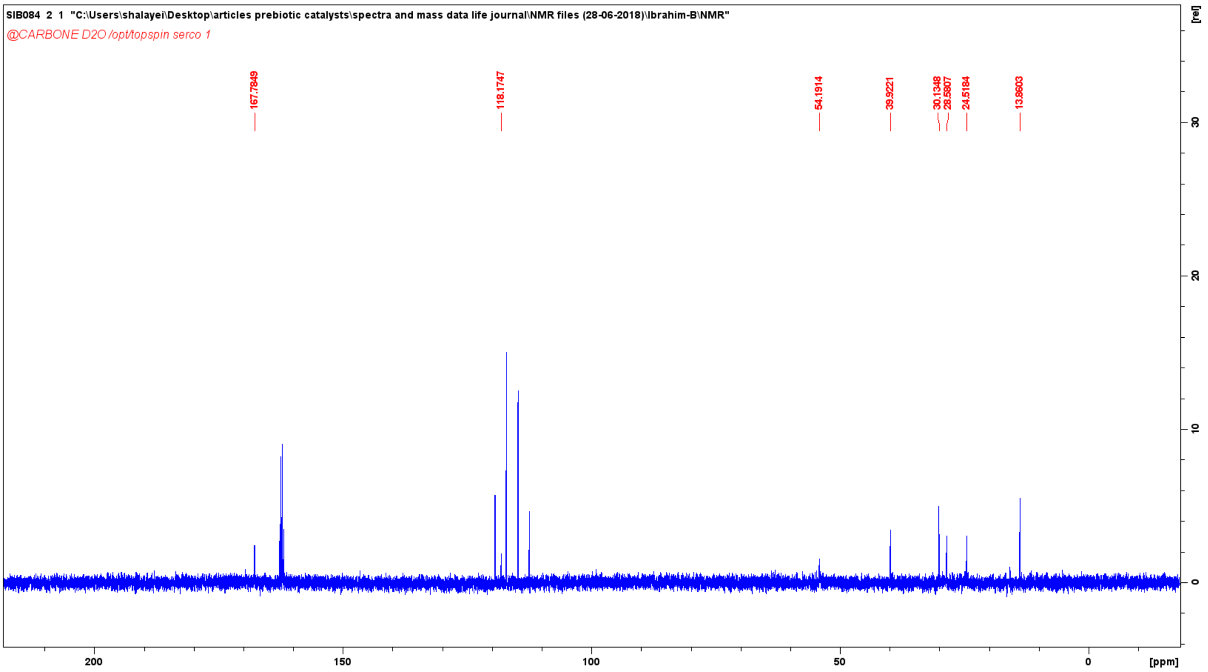

MALDI MS Spectrum for poly(Cys-Met)

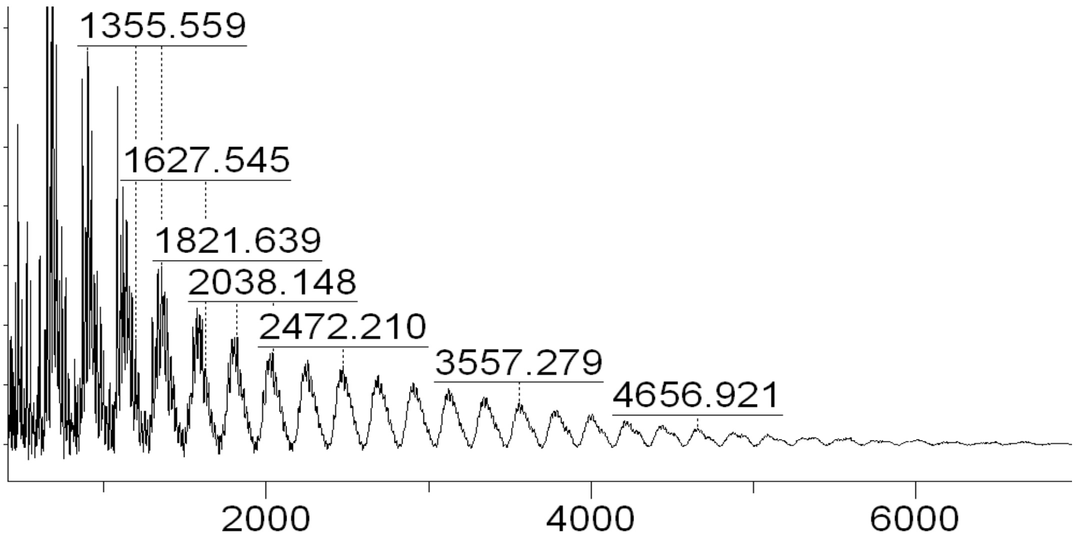

Synthesis and analysis of the prepared molecules

**General method for the preparation of methyl esters 3 and 4:**

Boc-L-Cys(Trt)-OH (1 equiv.) was dissolved in dry THF (~ 40 mL) at -20 °C under argon. *N*-methyl morpholine (NMM; 3 equiv.) and *iso*butyl chloroformate (IBCF; 1.5 equiv.) were added and the reaction left to stir for 1 h. Then, ester Met-OMe **1** or Ala-OMe **2** (1.1 equiv.) was added and the mixture was left stirring under argon overnight at rt. The mixture was concentrated, quenched with water and extracted three times with DCM. The organic phase was dried over $Na_2SO_4$ and evaporated under reduced pressure. Flash chromatography afforded the pure esters **3** and **4.**

**(*S*)-methyl 2-((*R*)-2-((*tert*-butoxycarbonyl)amino)-3-(tritylthio)propanamido)-4-(methylthio)butanoate (3)**

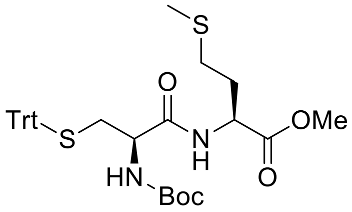


Following the general procedure, Boc-L-Cys(Trt)-OH (3 g, 6.48 mmol) was dissolved in THF. NMM (2.14 mL, 19.44 mmol) and IBCF (1.26 mL, 9.72 mmol) were added to give the mixed anhydride. Met-OMe (1.294 g, 6.48 mmol) was added. The crude product was submitted to silica gel column chromatography (90-80 % pentane/EA). The solvent was collected and concentrated under vacuum to give **3** as a white solid (59 %).

**m.p.** 121.9-123.2 °C; **$[\alpha]^{20}_D$** +25.7 (*c* 1.03, $CHCl_3$); **$^1$H-NMR (400 MHz, $CDCl_3$) (δ, ppm)**: 7.41 (d, J = 7.7 Hz, 6H), 7.25 (t, *J* = 7.6 Hz, 6H), 7.17(t, *J* =7.2 Hz, 3H), 6.85 (d, *J* = 7.0 Hz, 1H), 5.07 (d, *J* = 6.0 Hz, 1H), 4.63 (dd, *J* =12.6, 7.4 Hz, 1H), 3.86 (s ,1H), 3.64 (s, 3H), 2.78 (dd, *J* = 12.6, 7.2 Hz, 1H), 2.53 (dd, *J* = 12.6, 4.9 Hz, 1H), 2.46 (t, *J* = 7.4 Hz, 2H), 2.09-2.18 (m, 1H), 1.99 (s, 3H), 1.88-1.97 (m, 1H), 1.41 (s, 9H); **$^{13}$C-NMR (100 MHz, $CDCl_3$) (δ, ppm):** 171.81, 170.44, 155.29, 144.45, 129.57, 128.09, 126.91, 80.09, 67.14, 52.39, 51.51, 31.63, 29.74, 28.32, 15.34; **HRMS** (ESI): calcd for $C_{33}H_{40}N_2O_5S_2Na$ [M + Na]$^+$: 631.2276, found 631.2278.

**(*S*)-methyl 2-((*R*)-2-((*tert*-butoxycarbonyl)amino)-3-tritylthio)propanamido)propanoate (4)**

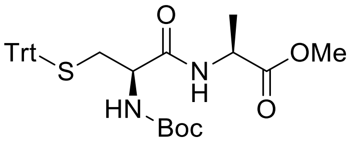

Following the general procedure, Boc-L-Cys(Trt)-OH (1.5 g, 3.24 mmol) was dissolved in THF. NMM (1.07 mL, 9.72 mmol) and IBCF (0.63 mL, 4.6 mmol) were added to give the mixed anhydride. Ala-OMe (0.452 g, 3.24 mmol) was added. The crude product was submitted to silica gel column chromatography (95-80 % pentane/EA). The solvent was collected and concentrated under vacuum to give **4** as a white solid (80 %).

**m.p**. 195.4-196.5 °C; **$^1$H-NMR (400 MHz, $CDCl_3$) (δ, ppm):** 7.35 (d, *J* = 7.5 Hz, 6H), 7.23 (t, *J* = 7,5 Hz, 6H), 7.16 (dd, *J* = 13.2, 7.2 Hz, 3H), 6.44 (br, 1H), 4.69 (br, 1H), 4.42 (quin, *J* = 7.2 Hz, 1H), 3.75 (br, 1H), 3.63 (s, 3H), 2.60-2.73 (b ,1 H), 2.45 (dd, *J* = 12.9, 4.9 Hz, 1H), 1.35 (s, 9 H), 1.28 (d, *J* = 7.1 Hz, 3H); **$^{13}$C-NMR (100 MHz, $CDCl_3$) (δ, ppm)**: 172.84, 169.93, 155.32, 144.41, 129.60, 128.09, 126.92, 80.27, 67.22, 53.49, 52.37, 48.13, 33.65, 28.22, 18.94.

**General method for the preparation of acids 5 and 6:**

Ester **3** or **4** (1 equiv.) was dissolved in a THF/water solution (2/1). LiOH (2.5 equiv.) was added to the mixture at 0 °C. The mixture was left to stir for 2 h at rt. The solution was concentrated and water was added. Then, $NaHSO_4$ (sat.) was added to afford pH 2-3. The mixture was extracted three times with DCM. The organic layer was collected, dried over $Na_2SO_4$ and concentrated under reduced pressure. Flash chromatography afforded the pure acids **5** and **6**.

**(*S*)-2-((*R*)-2-((*tert*-butoxycarbonyl)amino)-3-(tritylthio)propanamido)-4-(methylthio)butanoic acid (5)**

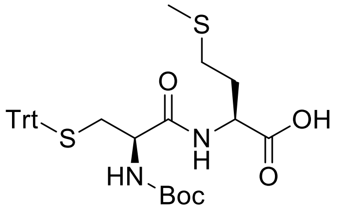

Following the general procedure, LiOH (399 mg, 9.5 mmol) was added to a solution of ester **3** (2.4 g, 3.80 mmol). The crude product was submitted to silica gel column chromatography (90-10 % pentane/EA). The solvent was collected and concentrated under vacuum to give **5** as a white solid (74 %).

**m.p.** 56.2-58.2 °C; **$[\alpha]^{20}_{D}$** +6.474 (*c* 1.00, $CHCl_3$); **$^1$H-NMR (400 MHz, $CDCl_3$) (δ, ppm)**: 10.39 (br, 1H), 7.41 (d, *J* = 7.7 Hz, 6H), 7.29 (t, *J* = 7.7 Hz, 6H), 7.21(t, *J* =7.3 Hz, 3H ), 6.85 (s, 1H,), 4.86 (s, 1H), 4.62 (d, J = 4.4 Hz, 1H), 3.87 (dd, *J* = 12.3, 7.1 Hz, 1H), 2.77 (dd , *J* = 13.0, 7.1 Hz, 1H), 2.51 (dd, *J* = 13.0, 5.0 Hz, 1H), 2.49 (t, *J* = 7.3 Hz, 2 H), 2.16-2.23 (m, 1H), 2.03 (s, 3H), 1.94-2.01 (m, 1H), 1.41 (s, 9H); **$^{13}$C-NMR (125 MHz, $CDCl_3$) (δ, ppm)**: 174.72, 170.74, 155.59, 144.36, 129.56, 128.13, 126.95, 80.75, 67.28, 53.60, 51.81, 33.37, 31.42, 29.75, 28.29, 15.34; **HRMS** (ESI): calcd for $C_{32}H_{38}N_2O_5S_2Na$ $[M + Na]^+$: 617.2120, found 617.2120.

**(*S*)-2-((*R*)-2-((*tert*-butoxycarbonyl)amino)-3-(tritylthio)propanamido)propanoic acid (6)**

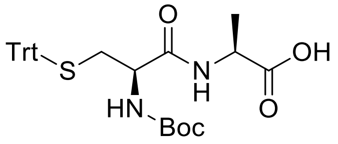

Following the general procedure, LiOH (178 mg, 4.24 mmol) was added to a solution of ester **4** (930 mg, 1.69 mmol). The crude product was submitted to silica gel column chromatography (90-30 % pentane/EA). The solvent was collected and concentrated under vacuum to give **6** as a white solid (85 %).

**m.p.** 80.7-81.2 °C; **$[\alpha]^{20}_{D}$** +10.38 (*c* 1.05, $CHCl_3$): **$^1$H-NMR (400 MHz, $CDCl_3$) (δ, ppm)**: 7.35 (d, *J* = 7.7 Hz, 6H), 7.22 (t, *J* = 7.7 Hz, 6H), 7.16 (t, *J* = 7.3, 3H), 6.59 (d, *J* = 5.5 Hz, 1H), 4.75 (br, 1H), 4.41 (quin, *J* = 6.8 Hz, 1H), 3.76 (br, 1H), 2.66 (dd, *J* = 12.8, 7.2 Hz, 1H), 2.47 (dd, *J* = 12.8, 4.7 Hz, 1H), 1.34 (s, 9 H) 1.32 (d, *J* = 7.5 Hz, 3H); **$^{13}$C-NMR (100 MHz, $CDCl_3$) (δ, ppm)**: 174.01,

170.50, 155.85, 144.19, 129.49, 128.19, 127.07, 80.91, 67.47, 54.00, 48.57, 33.28, 28.34, 17.54.

**General method for the preparation of amides 7 and 8:**

Acid **5** or **6** (1 equiv.) was dissolved in dry THF at -20 °C under argon. NMM (2 equiv.) and IBCF (1.5 equiv.) were added and the reaction left to stir for 30 min. Then, ammonia gas was bubbled into the solution for 10 minutes and the mixture was left stirring under argon overnight at rt. The mixture was concentrated, quenched with water and extracted three times with EA. The organic phase was dried over $Na_2SO_4$ and evaporated under reduced pressure. Flash chromatography afforded the pure amides **7** and **8**.

***tert*-butyl ((*R*)-1-(((*S*)-1-amino-4-(methylthio)-1-oxobutan-2-yl)amino)-1-oxo-3-(tritylthio)propan-2-yl)carbamate (7)**

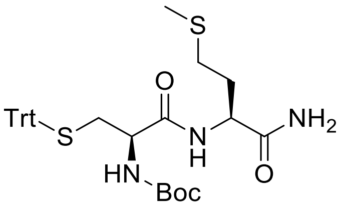


Following the general procedure, acid **5** (1.25 g, 2.03 mmol), NMM (355 µL, 3.05 mmol) and IBCF (526 µL, 4.06 mmol) were used. The crude product was submitted to silica gel column chromatography (90-50 % pentane/EA). The solvent was collected and concentrated under vacuum to give **7** as a white solid (81 %).

**m.p.** 85.3-86.9 °C; **$[\alpha]^{20}_D$** –10,88 (*c* 1.00, $CHCl_3$); **$^1$H-NMR (400 MHz, $CDCl_3$) (δ, ppm)**: 7.41 (d, *J* = 7.5 Hz, 6H), 7.31 (t, *J* = 7.5 Hz, 6H), 7.24 (t, *J* =7.2 Hz, 3H ), 6.92 (s, 1H), 6.66 (s, 1H), 5.27 (s, 1H), 4.72 (s, 1H), 4.55-4.61 (m ,1 H), 3.77 (dd, *J* = 11.3, 5.2 Hz, 1H), 2.78 (dd, *J* = 12.9, 6.7 Hz, 1H), 2.58 (dd, *J* =12.9, 4.9 Hz, 1H), 2,11-2,23 (m, 1H), 2.04 (s, 1H), 1,95-2.10 (m, 1H), 1.42 (s, 9 H); **$^{13}$C-NMR (100 MHz, $CDCl_3$) (δ, ppm)**: 173.23, 170.52, 155.84, 144.20, 129.48, 128.18, 127.06, 80.85, 67.40, 60.41, 54.13, 52.24, 33.27, 28.27, 15.19; **HRMS** (ESI): calcd for $C_{32}H_{39}N_3O_4S_2Na$ $[M + Na]^+$: 616.2280, found 616.2277.

***tert*-butyl ((*R*)-1-(((*S*)-1-amino-1-oxopropan-2-yl)amino)-1-oxo-3-(tritylthio)propan-2-yl)carbamate (8)**

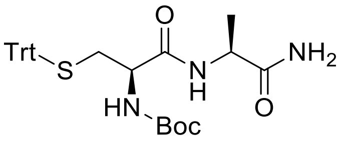


Following the general procedure, acid **6** (595 mg, 1.11 mmol), NMM (0.19 mL, 1.7 mmol) and IBCF (0.29 mL, 2.23 mmol) were used. The crude product was submitted to silica gel column chromatography (90-50 % pentane/EA). The solvent was collected and concentrated under vacuum to give **8** as a white solid (88 %).

**m.p.** 97.3-101.2 °C; **$[\alpha]^{20}_{D}$** –8.0 (*c* 0.65, $CHCl_3$); **$^1$H-NMR (400 MHz, $CDCl_3$) (δ, ppm)**: 7.42 (d, *J* = 7.6 Hz, 6H), 7.30 (t, *J* = 7.5 Hz, 6H), 7.24 (t, *J* = 7.2 Hz, 3H), 6.47 (s, 1H), 6.31 (d, *J* =7.7 Hz, 1H), 5.21 (s, 1H), 4.74 (d, *J* = 3.7 Hz, 1H), 4.43 (quin, *J* = 7.3 Hz, 1H), 3.75 (d, *J* = 4.9 Hz, 1H), 2.71 (dd, *J* = 12.8, 7.4 Hz, 1H), 2.61 (dd, *J* = 12.8, 5.3 Hz, 1H), 1.42 (s, 9 H) 1.36 (d, *J* = 7.2 Hz, 3H); **$^{13}$C-NMR (100 MHz, $CDCl_3$) (δ, ppm)**: 174.06, 170.29, 155.89, 144.19, 129.46, 128.19, 127.07, 80.91, 67.47, 53.98, 48.57, 33.28, 28.34, 28.23, 17.55.
